# Functional Coatings or Films for Hard-Tissue Applications

**DOI:** 10.3390/ma3073994

**Published:** 2010-07-09

**Authors:** Guocheng Wang, Hala Zreiqat

**Affiliations:** Biomaterials and Tissue Engineering Research Unit, School of AMME, The University of Sydney, Sydney 2006, Australia; E-Mail: guocheng.wang@sydney.edu.cn

**Keywords:** metal, corrosion, wear, bioactivity, biocompatibility, antibacterial, surface modification

## Abstract

Metallic biomaterials like stainless steel, Co-based alloy, Ti and its alloys are widely used as artificial hip joints, bone plates and dental implants due to their excellent mechanical properties and endurance. However, there are some surface-originated problems associated with the metallic implants: corrosion and wear in biological environments resulting in ions release and formation of wear debris; poor implant fixation resulting from lack of osteoconductivity and osteoinductivity; implant-associated infections due to the bacterial adhesion and colonization at the implantation site. For overcoming these surface-originated problems, a variety of surface modification techniques have been used on metallic implants, including chemical treatments, physical methods and biological methods. This review surveys coatings that serve to provide properties of anti-corrosion and anti-wear, biocompatibility and bioactivity, and antibacterial activity.

## 1. Introduction

Metallic biomaterials like stainless steel, Co-based alloy, Ti and Ti alloys are widely used as artificial hip joints, bone plates and dental implants due to their excellent mechanical properties and endurance [1]. However, long-term performance of surgical implants is directly depending on their surface properties. Most implanted metallic biomaterials have a tendency to lose electrons in solution and, as a result, they show a high potential to corrode in the biological environments, which usually cause inflammatory and loosening of the implants [2]. Additionally, their low surface hardness, high friction coefficient and poor wear resistance are also limiting their application of metallic biomaterials [3,4]. It is reported that wear and corrosion are the main reasons for degradation of surgical implants such as hip and knee joint implants, which usually happens after 10–15 years of use [4]. Another problem associated with metallic implants is their biological inertness. Bioinert materials are incapable of inducing positive connective osteogenesis or new bone ingrowth, thus only low fixation strength can be achieved between the implant and the host bone [5]. To protect the metallic implants from corrosion and wear and improve their bioactivity, tremendous surface modification techniques have been applied to deposit a great variety of functional coatings on the surfaces of metallic implants. As the implant-related bacterial infection remains a major impediment to the utility of medical implants despite of the use of sterilization and aseptic techniques, researchers are also endeavoring to develop coatings with antibacterial activity [6].

**Figure 1:**
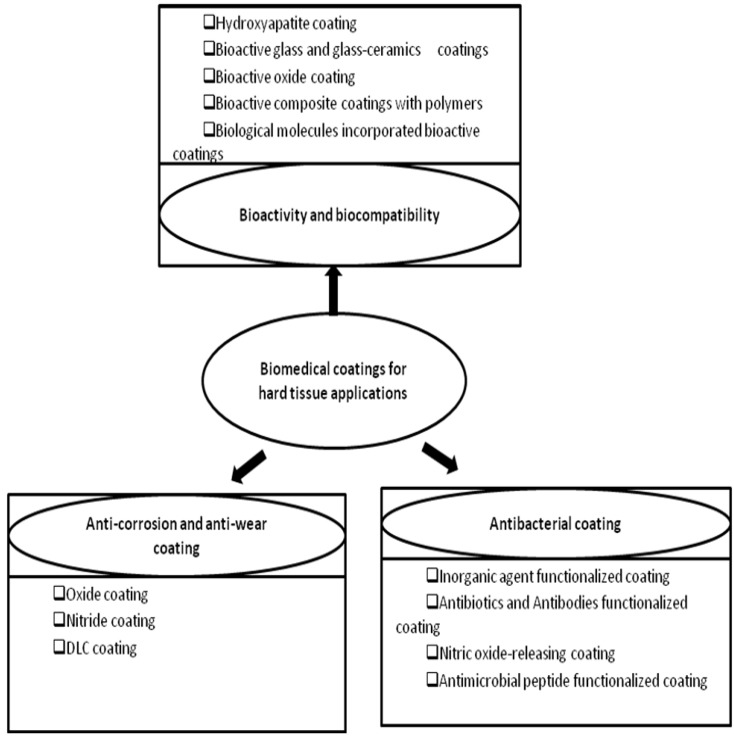
The outline of this review paper.

This review is aiming to give a comprehensive summary of these coatings. The outline of this paper is displayed in Figure 1. Since there have been already many review papers about the surface modification methods, this review primarily focus on the functions of the coatings as well as their influencing factors, instead of basic knowledge of surface modification techniques. For basic knowledge of the techniques mentioned in this article, readers can refer to related references cited there.

## 2. Anti-corrosion and Anti-wear Coatings

Metals are widely used as biomaterials due to their low thermal conductivity, high ductility and excellent combination of high strength and low modulus. Presently, the materials used for this application are 316 L stainless steel, cobalt chromium alloy, Ag, Au, and titanium-based alloys as well as magnesium alloys. Some of them are used as passive substitutes for hard tissue replacement such as total hip and knee joints, fracture healing aids as bone plates and screws, spinal fixation devices, and dental implants. Some other alloys are used for more active roles in devices such as vascular stents, catheter guide wires, orthodontic archwires, and cochlea implants [7]. There are also some metals for specialized implant application. For example, radioactive tantalum (Ta182) has been used to treat head and neck tumors [8].

However, most implanted metals, such as titanium, cobalt-chromium and stainless steels, have a tendency to lose electrons in solution and, as a result, they show a high potential to corrode. Corrosion is the unwanted chemical reaction, which can result in degradation of metal implants to oxides, hydroxides, or other compounds. These degradation products may cause a local inflammatory response, finally lead to the cessation of bone formation, synovitis, and loosening of artificial joint implants [2]. The service period of the material is mainly determined by its abrasion and wear resistance. The low wear resistance can lead to the formation of wear debris which may cause several reactions in the tissue in which they are deposited thus increasing failure probability of the implants. Another detrimental aftermath of metal corrosion and wear is that it can weaken an implant, causing premature failure [9]. Therefore, there is a significant need to design or invent metal implants with enhanced corrosion and wear resistance.

There are two usual ways to improve the corrosion and wear resistance of a metal implant. One is via bulk alloying and the other is via surface modification. Since this paper only focuses on surfaces of the metal implants, the former is not covered here. In this section, coatings for anti-corrosion and anti-wear are divided into several groups: oxide coatings; nitride coatings and diamond-like carbon coatings. The related surface techniques are also briefly introduced.

### 2.1. Oxide Coatings

#### 2.1.1. Thermal oxidation technique (TO)

Thermal oxidation technique is a widely used novel surface engineering process to improve the anti-corrosion and anti-wear properties of Cp Ti and its alloy [10,11,12,13,14,15,16,17,18,19], based on thermochemical reaction. It is usually carried out in a controlled atmosphere containing oxygen and nitrogen at approximately 600 °C [18]. Borgioli *et al.* [13] successfully fabricated oxide layer on the Ti-6Al-4V by treating the Ti-6Al-4V in an air circulating furnace at 1173 K for 2 h at 105 Pa followed by quenching using compressed air. Wear tests, carried out on both untreated and oxidized samples in block-on-ring test configuration under dry sliding conditions, showed that thermal oxidation treatment was able to substantially improve the wear resistance of Ti-6Al-4V samples, reducing the wear volumes by about 4 to 6 times in comparison with the untreated alloy. The improved performance was due to the formation of the hard oxide layer and the oxygen diffusion zone (ODZ) beneath it [12]. The Vickers hardness was increased from 169 to 492 (100 g force) for Cp-Ti and 369 to 755 (100 g force) for Ti-6Al-4V after thermal oxidation treatment [12]. Both the oxide layer and ODZ contributed to the improvement of the wear resistance and the prevention from extensive corrosion-wear of the Ti-6Al-4V [12,19]. However, the oxide layer produced by thermal oxidation, especially at high temperatures (above 800 °C) and with prolonged soaking times, has a low bonding strength with the substrates and is inclined to debonding [20]. To solve this problem, cooling treatments with a very slow rate can be applied on titanium after thermal oxidization [10]. As far as biological properties, it was demonstrated that Ti and Ti-6Al-4V alloy showed improved biocompatibility after thermal oxidation [11,21].

#### 2.1.2. Microarc oxidation (MAO)

Microarc oxidation, also called microplasma oxidation or anodic spark deposition, is a surface modification technology developed to produce hard, thick oxide coating on metallic substrates. Xue *et al.* [22] fabricated ceramic coatings on Ti-6Al-4V by micro-arc oxidation in aluminate solution. The coating was mainly composed of TiO_2_ in rutile form and TiAl_2_O_5_ compounds. The content of TiO_2_ increased from the surface to the interior of the coating while the TiAl_2_O_5_ was quite the reverse. Nanoindentation tests showed that the hardness was significantly improved by MAO and gradually increased from the surface to the interior consistent with the variation of TiO_2_ fraction along the coating thickness. The kinetics of MAO coatings is interface-controlled and largely dependent on the applied current density and treatment time [23,24].

Besides Ti and Ti alloys, MAO has been also widely used on magnesium alloy to protect it from corrosion and wear [24,25,26,27,28]. The corrosion resistance of AZ91D magnesium was improved over 100 times after MAO treatment in solution containing aluminate and potassium fluoride at constant applied current densities, due to the formation of the ceramic coating composed of spinel MgAl_2_O_4_ phase and intermetallic Al_2_Mg phase [25]. However, MAO coating generally possesses a foam-like structure with high bulk porosity, which is undesired in anti-corrosion and anti-wear applications. The work done by Guo *et al.* [29] showed that the addition of surfactants in the electrolyte could successfully inhibit the generation of pores within ceramic coatings, thus improving the coating quality. Silicate and phosphate electrolytes are also often used in MAO process, as coating fabricated from both these two electrolytes could protect magnesium from corrosion and wear [28,30,31]. But coatings formed from silicate solution showed better anti-corrosion ability than that from phosphate solution due to their different phase composition and coating structure [28,30]. Coatings formed from silicate electrolyte are usually compact and are mainly composed of Mg_2_SiO_4_ and MgO, while coatings from phosphate are relatively porous and are mainly composed of MgO.

#### 2.1.3. Oxygen ion implantation

Oxygen ion implantation can also be used to improve the wear and corrosion resistance as well as biocompatibility of the metallic materials. Ion implantation includes conventional beam-line ion implantation and plasma immersion ion implantation (PIII). In the conventional beam-line ion implantation, an ion source is used to create an ion beam of the species to be implanted, and then the ion beam is accelerated through high potential and bombarded into the substrates. It is a line-of-sight process so that the objects must be correctly manipulated to get the desired surface implanted. PIII initially developed by Conrad can circumvent the beam line restrictions and does not require the manipulation of the substrates [32]. It has been widely used to improve the surface properties of biomedical metal materials, such as wear resistance, corrosion resistance and biocompatibility.

Leng *et al.* [33] demonstrated that plasma immersion ion implantation of titanium and oxygen on Ti-6Al-4V greatly improved the wear resistance of the Ti-6Al-4V under low load. The hardness of the newly formed TiO_x_ film increased with increasing oxygen partial pressure in the range of 0–3 × 10^-2^ Pa, and reached the maximum of 17 GPa at an oxygen partial pressure of 3 × 10^-2^ Pa. However, the thickness of the films was suboptimal for this application. The thickness of the surface layer was observed to depend on the implantation temperature and treatment time [34]. At low temperature, oxygen does not adequately diffuse into the bulk material thus limiting the layer thickness. For NiTi alloys, Tan *et al.* [35] demonstrated that the thickness of the oxide coating using oxygen implantation was influenced by the austenite finishing transformation temperature (*A_f_*). In their work, aging treatments followed by quenching were performed on commercial Ti-50.7 at.% Ni alloys before ion implantation. The *A_f_* of sample treated at 550 °C for 20 min and 400 °C for 70 min were −3° and 21°, respectively. The oxide thickness was found to be 1,140 nm for *A_f_* = 21° samples which was 370nm higher than that of *A_f_* = −3° samples. One reason for this was thought to be the different holding time at elevated temperature. Additionally, the presence of martensite increased the average oxygen penetration because this phase was dilated with respect to austenite. The fact that samples with *A_f_* = 21° are more inclined to experience austenite-to-martensite phase transformation near room temperature, might be another important reason for the thicker oxide on the samples [35]. Their further study showed that samples with *A_f_* = 21° and oxygen implanted at a dose of 1 × 10^17^ ions/cm^2^ had the best pitting corrosion resistance. Increasing the oxygen dose to 3 × 10^17^ ions/cm^2^ resulted in the formation of nano pores which impaired the pitting corrosion resistance [36]. Fretting wear tests followed by measuring the wear scar volume showed that oxygen implantation improved the wear resistance of NiTi alloys [37].

#### 2.1.4. Sol-gel method

Sol-gel derived oxide films or coatings like SiO_2_, Al_2_O_3_, TiO_2_ and ZrO_2_, can be also deposited on the metals to improve their resistance to corrosion and wear. The sol-gel process, also known as chemical solution deposition, is a wet-chemical technique, a process involving five main steps: (1) hydrolysis and polycondensation; (2) gelation; (3) aging; (4) drying; (5) densification and crystallization. Compared to conventional thin film processes, sol-gel process allows for better control of the chemical composition and microstructure of the films utilizing simple equipments at low cost. Especially, the heat treatment temperature it needs is lower because precursors can be mixed at molecular level in the solution and thus a high degree of homogeneity can be obtained in the films.

Sol-gel thin coatings of ZrO_2_, SiO_2_, 70SiO_2_-30TiO_2_ and 88SiO_2_-12Al_2_O_3_ composition ( mol %) have been prepared from sono-catalyzed sols and deposited by dip-coating technique on 316 L stainless steel foils [38]. All of the coatings resulted in a lower corrosion rate compared to the uncoated samples. Liu *et al.* [39] deposited TiO_2_ coatings on NiTi alloy using sol-gel method and compared their corrosion resistance and blood compatibility to the uncoated sampled. The coating was 205 nm in thickness and mainly composed of rutile TiO_2_ after sintered at 500 °C. Electrochemical tests on the coated and uncoated NiTi alloys in Tyrode’s solution showed an increase in the breakdown potential (E_b_) by 200 mV and a decrease in the passive current density (i_p_), indicating the improved corrosion resistance of the NiTi alloys by TiO_2_ coating. Furthermore, blood compatibility of the coated NiTi alloy was also superior to the uncoated metal. Tribological behavior of sol-gel TiO_2_ film on a glass substrate was studied by using a reciprocating friction and wear tester sliding against Si_3_N_4_ ball and AISI52100 steel [40]. It was demonstrated that the friction of the glass was highly reduced after coated with TiO_2_ film. A slight plastic deformation of the film and its good adhesion to the substrate were thought to be the main reasons for its improved wear resistance. Jia *et al.* [41] found that adding suitable amount of SiO_2_ to TiO_2_ films improved anti-wear and friction-reduction performance of TiO_2_ film, which was ascribed to the reduction in TiO_2_ grain size, the increase in adhesion strength and the formation of Si-O-Ti hetero-linkages. Consequently, the wear mechanism changed from plastic deformation and abrasive wear to light scuffing and abrasion, which was the same as the sol-gel TiO_2_ films with well-dispersed Ag particles [41]. However, it was not the case when the SiO_2_ film was used alone or as an interlayer, instead of as a dopant. Zhang *et al.* [42] systematically studied the anti-wear properties of SiO_2_, TiO_2_ and hydroxyapatite (HA) films on Ti-6Al-4V prepared by sol-gel methods. The wear resistance of SiO_2_ was the worst both under low load (1 N) and high load (3 N). However, a worthy finding was that sol-gel HA films had the best wear resistance under both low and high load. Their further study on TiO_2_, SiO_2_, HA, TiO_2_-HA and SiO_2_-HA thin films showed that the wear resistance of the HA dual films (TiO_2_–HA and SiO_2_–HA) deteriorated both under 3 N and 1 N due to residual stress in the dual films caused by the difference of thermal expansion coefficient between HA and TiO_2_ or SiO_2_ [43]. But under 0.5 N loads, a longer wear life was obtained for TiO_2_-HA films, due to the insufficiency of the load to induce the release of the internal stress between the films. Besides the TiO_2_, ZrO_2_, SiO_2_ and HA mentioned above, Al_2_O_3_ sol-gel films also showed good wear resistance, but are rarely used as anti-wear coating for biomedical metal due to its possible cytotoxicity [44].

#### 2.1.5. Thermal spraying technique

Thermal spraying is also a useful coating technique for enhancing the corrosion and wear resistance of biomedical metallic implants. Briefly, thermal spraying is a process in which melted or semi-melted particles are sprayed onto a substrate surface. It can be divided into many categories according to energy sources used for heating or melting the powder particles, such as plasma spraying, flame spraying, high velocity oxy-fuel spraying, arc spraying and so on.

Due to its capacity for ultrahigh temperature heating and fabricating components with alternate layers of different material composition as well as low operating and capital cost, plasma spray has drawn lots of attentions in many fields. A wide variety of ceramic coatings, such as Cr_2_O_3_-SiO_2_-TiO_2_, Cr_2_O_3_, Cr_2_O_3_-Ni-Cr, WC-Co, TiO_2_, ZrO_2_, Al_2_O_3_ and like that, have been studied on their tribological properties [45]. However, only TiO_2_ [46,47], Al_2_O_3_ [48] and ZrO_2_ [49] coatings have been tried to be used as biomedical coating due to their good biocompatibility. The recent progress in plasma sprayed anti-wear coatings are summarized as follows:

A Composite with other materials: Al_2_O_3_ is an attractive material for wear-resistance due to its high hardness and high thermal conductivity. However, brittleness is its main problem limiting its application in some fields. The addition of TiO_2_ and ZrO_2_ can improve the fracture toughness of Al_2_O_3_ but also lower its hardness [50,51]. Optimizing the appropriate proportion of alumina and zirconia to achieve a composite with improved wear resistance remains to be a challenge.
B Nanostructured coatings: Grain size is another important factor influencing the wear resistance of materials. The relationship between wear resistance and the grain size follows the type of Hall-Petch law [52]. When the grain size is reduced to nanosize, higher external stress is required to induce grain boundary cracking and pulling-out of grains, hence, the nanostructured coatings shows better plastic deformation ability than traditional coating during sliding wear. Moreover, an improved hardness and toughness are also observed for the nanostructured coating [53]. Therefore, nanosized grains are expected to be able to improve the wear resistance of the coatings [54]. Chen *et al.* [55] compared the friction and wear properties of plasma sprayed nanostructured and conventional zirconia coating against stainless steel with a sliding, reciprocating and vibrating test machine under water-lubricated conditions. It was found that plasma sprayed nanostructured zirconia coatings possessed better wear resistance than traditional coatings in that the wear rate of the former was in the range from one-fourth to four-fifths of the latter under loads ranging from 20 to 50N. The great effects of nanostructured coating on wear resistance are also improved by other researchers using other coating systems like TiO_2_ [47], Al_2_O_3_-ZrO_2_ [56] and Al_2_O_3_-TiO_2_ [57].
C Post-treatments: There are two major problems with plasma spraying. The primary problem is the poor bonding strength between the coating and substrates, which causes the sprayed material to peel off under high bending stress. The second problem is the high porosity of the coating, which usually reduces the anti-corrosion and anti-wear performance. To overcome these drawbacks, post-treatments by laser are often used. Post-treatments like laser remelting can significantly reduce the porosity and roughness of plasma sprayed coating, and apparently improve the bonding strength, thus enhancing the wear resistance of the as-sprayed coating [58]. For plasma sprayed zirconia coating, laser remelting could change the main wear mechanism from spallation to ploughing and gouging [58]. For Al_2_O_3_-13 wt. % TiO_2_ coating, the enhanced wear resistance after laser melting were ascribed to not only the improvement of the microstructures, but also the transformation from metastable phase γ-Al_2_O_3_ to stable phase α-Al_2_O_3_ [59].

### 2.2. Nitride Coatings

ZrN coating or film has been drawing attentions for its excellent erosion resistance, biocompatibility, high hardness, good lubricity and ductility. Its corrosion resistance has been studied on many different metal substrates, including AZ91 Mg alloys [60], Ni-Ti shape memory alloy [61], AISI 304 stainless steel [62] and Ti6Al4V alloy [63]. Table 1 is a summary of the anti-corrosion properties of ZrN coating or film deposited on different metal substrates using different methods.

Xin *et al.* [60] deposited ZrN/Zr coatings on biodegradable magnesium alloys using a filtered cathodic arc deposition system to inhibit its degradation in aqueous environment. Electrochemical tests in simulated body fluid (SBF) showed that the corrosion potential of the uncoated alloy was quite negative and only about −1830 mV while that of the coated alloy was much more positive, shifting to about −1420 mV. The corrosion current density of the coated alloy was also significantly improved, with about two orders of magnitude lower than that of the uncoated alloy. These results indicated the corrosion resistance was significantly improved by ZrN coating. In this paper, the Zr interlayer was designed to buffer the mismatch of the ZrN coating and the magnesium alloy, thus resulting in enhanced adhesion strength. However, this layer is also able to contribute to the corrosion resistance, Chou *et al.* [64] reported that bi-layer ZrN/Zr coating deposited on 304 stainless steel by a hollow cathode discharge ion plating (HCD-IP) system exhibited the highest corrosion resistance in comparison with single-layered Zr and ZrN coatings. The mechanism was explained as follows: the corrosion resistance of the specimen significantly dependent on the pinhole number and size [64], since the corrosion occurred via the diffusion of the electrolyte through pinholes and attacking the underneath metal substrates. The Zr layer was suggested to interrupt the pinhole connection through the coating surface to the underlying substrate, therefore reducing the exposure area of the substrate to the electrolyte.

**Table 1:** Summary of the anti-corrosion ZrN coatings.

Composition	Substrates	Methods	Electrolyte	Ref.
ZrN/Zr	biomedical AZ91 magnesium alloy	filtered cathodic arc deposition	simulated body fluids (SBF)	[60]
Zr, ZrN and ZrN/Zr	AISI 304 stainless steel	Hollow cathode discharge ion plating	0.5 M H_2_SO_4_ containing 0.05M KSCN	[64]
ZrN0.83/Zr	NiTi shape-memory alloy	plasma immersion ion implantation and deposition	Hank’s Solution	[75]
ZrN,TiN and Ti/TiN	316 L stainless steel	reactive magnetron sputtering	pH 5.6 acetic acid and sodium acetate buffer solution.	[62]
ZrN and ZrN-Ag nanocomposite	AISI 316 L surgical steel, and medical grade Ti-Al-V	reactive unbalanced magnetron sputtering	3.5% NaCl solution	[65]
TiN and ZrN	Plain carbon steel	an unbalanced magnetron sputtering technique/low or mild energetic ion bombardment with high flux	sulfuric acid solution (1N)	[66]

More interestingly, the incorporation of a certain amount of silver (Ag) in the ZrN coating enhanced the corrosion resistance of the coating whilst also introducing antibacterial properties to the coating. Kertzman *et al.* [65] reported that the nanocomposite films of ZrN-Ag fabricated using reactive unbalanced magnetron sputtering possessed a dense and homogenous microstructure wherein Ag nanocrystals were distributed evenly throughout the ZrN matrix. Its corrosion resistance was proved to be better than ZrN coating alone and dependent on the bias potential used during deposition. It was thought that Ag addition reduced the depth of the pits distributed in the coating, thus retarding the pitting corrosion. The effect of surface defects or morphologies on the corrosion resistance was investigated by Kelesoglu *et al.* [66]. They found that the improved corrosion resistance of the magnetron sputtered ZrN coating on plain carbon steels (Ck35) was parallel to the morphological improvement in the coatings (*i.e.*, reduced porosity and surface defects). Low or mild energetic ion bombardment with high flux was proved to be an effective way to improve the morphology of the coating.

Titanium nitride (TiN) is another important ceramic coatings being studied for a biomedical application as it has good biocompatibility [67,68], anti-corrosion and anti-wear properties [62,66,69,70,71,72,73,74]. Table 2 shows a summary of TiN coatings for anti-corrosion. TiN coating on the femoral head of Ti-6Al-4V artificial hip joints was proved to significantly reduce the passive current density by approximately 2 orders of magnitude [71] indicating great improvement of the anti-corrosion property. However, due to some inherent shortcomings of some film deposition methods, or impropriety of the process parameters, surface defects like pinhole or macro pore, sometimes even micro cracks are often formed in the coating. In this case, although the corrosion rate can be somewhat reduced in the early stage, it deteriorates rapidly after the long-term immersion in electrolyte as pitting corrosion occurs in the pinhole [70].

**Table 2:** Summary of anti-corrosion TiN coatings.

Ref.	Substrate	Methods	Electrolyte
[62]	316-L stainless steel	reactive magnetron sputtering	pH 5.6 acetic acid and sodium acetate buffer solution
[66]	Plain carbon stee (Ck35)	unbalanced magnetron sputtering technique	1 N sulfuric acid solution
[69]	Biomedical NiTi shape memory alloy	plasma immersion ion implantation and deposition (PIIID)	Hank’s solution
[70]	1Cr11Ni2W2MoV Martensitic stainless steel	hollow cathode ionic plating (HCIP)	0.5 mol/L NaCl and 1mol/L H_2_SO_4_ diluted aqueous solution
[71]	Ti-6Al-4V	plasma assisted electron beam PVD technique	0.5 N NaCl solution
[73]	NiTi coated Si	dc magnetron sputtering	1 mol/L NaCl solution
[74]	Biomedical AISI 316L stainless steel	arc ion plating	neutral Troyde’s simulated body fluid

Just as the Zr interlayer can enhance the corrosion resistance of ZrN coating [64], the Ti interlayer can enhance that of TiN coating [74]. Additionally, Li *et al.* [70] demonstrated that introduction of certain amount of Al in the TiN coating improved the long-term performance of TiN coating because the corrosion process was obstructed by the corrosion product of Al on the interface between the coating and substrate. Other methods like ion beam mixing [62] and high-flux ion bombardment [66] can also improve the corrosion resistance of the TiN coating.

Fu *et al.* [76] deposited TiN layer on TiNi film by co-sputtering a Ti0.5Ni0.5 target on silicon substrate first and then sputtering Ti target in Ar/N_2_ atmosphere. It was reported that TiN layer formed on the TiNi film significantly increased its hardness from 2.5 GPa to 9.3 GPa despite the fact that the TiN layer was only about 300nm. The scratching and sliding wear tests showed that the coefficient of friction, load bearing capacity and wear resistance of the TiNi films were effectively improved. Besides the methods mentioned above, other techniques such as ion implantation [77,78,79,80,81], PVD [71,82,83,84], plasma nitriding [85,86,87] and laser nitriding [88,89,90] can also fabricate nitride protective coatings or films on metal implants.

In summary, both ZrN and TiN films or coatings can successfully protect metal implants from corrosion. However, the anti-corrosion ability is strongly depending on the quality of the coating. Defects such as pinholes, pits and macro-pores in the coating are detrimental to its corrosion resistance. Besides, the interfacial adhesion between the coating and metal substrates also plays an important role. Therefore, optimum process parameters and proper post-treatments are required.

### 2.3. Diamond-like Carbon (DLC) Films

Diamond-like carbon (DLC) has a high wear and corrosion resistance, chemical inertness, high electrical resistivity, infrared-transparency, high refractive index and excellent smoothness. All these merits render it a good biomaterial for application in orthopedic, cardiovascular, contact lenses, or dentistry. Many surface modification techniques have been applied to produce DLC films with a variety of carbonaceous precursor materials, including ion beam deposition [91], plasma-assisted chemical vapor deposition (PACVD) [92], filtered cathodic vacuum arc (FCVA) [93], ion plating [94], plasma immersion ion implantation and deposition (PIIID) [95], magnetron sputtering [96], ion beam sputtering [97], pulsed laser deposition [98] and mass selected ion beam deposition [99,100]. Some excellent reviews on DLC films have been published in the past several decades [101,102,103,104,105,106]. Reviews written by Robertson [104], Bhushan [103], and Erdemir [101] described the deposition methods, deposition mechanisms, characterization methods, electronic structure, gap states, defects, doping, luminescence, field emission, mechanical properties and some application of DLC. Reviews written by Dearnaley [105], Grill [102] and Hauert [106] mainly focused on the biocompatibility and biomedical application of the DLC films. This section is attempting to sum up the major influencing factors on the tribological and corrosive properties of the DLC films.

The mechanical and tribological properties of a DLC film depend on the sp^3^/sp^2^ ratio, the amount of hydrogen in the films, and adhesion of the film to the substrate. The type of deposition techniques, processing parameters like precursor materials, kinetic energy of the carbon species prior to deposition, deposition rate and even the substrates conditions greatly influence the mechanical and tribological properties of the DLC film [103].

A sp^3^/sp^2^ ratio: Two types of carbon-carbon interatomic bond exist in the diamond-like carbon (DLC) films, one is sp^2^ hybridization, as in graphite; the other one is sp^3^ hybridization, as in diamond. The sp^3^/sp^2^ ratio in different DLC films varies significantly depending on the type of the applied deposition techniques and the used procedure parameters. Usually, films with a high proportion of sp^2^-bonded carbon atoms tend to be relatively soft and behave more like graphite during tribological tests, while films with more sp^3^-bonded carbons are more like diamond, and hence they are superhard and provide impressive tribological properties. The review written by Bhushan states that sp^3^/sp^2^ frictions are in the decreasing order for cathodic arc deposition, pulsed laser vaporization, direct ion beam deposition, plasma-enhanced chemical vapor deposition (PECVD), ion beam sputtering and DC/RF sputtering [103]. In this review paper, it was also proposed that the deposition of sp^3^-bonded carbon required the depositing species to have a kinetic energy in the order of 100ev or higher. Excess energy, such as that from substrate heating, is detrimental to the achievement of high sp^3^ friction.
B Hydrogen content: DLC films sputtered with the addition of H_2_ or derived from a hydrocarbon source, such as acetylene or methane possess a large amount of hydrogen in the films. It is interesting that there is still about 10% hydrogen present in the DLC films sputtered in 100% Ar by direct current (DC) magnetron sputtering [107]. Hydrogen causes the shift of C-C bonds from sp^2^ to sp^3^, and generation of a larger number of C-H bonds which relieve the internal stress and produces a softer polymer-like materials. Compared with hydrogen-free DLC films, such films with a high degree of hydrogenation have low friction and wear especially when tests are performed in inert or vacuum test environments [101]. But in the moisture or water environments, their friction increases substantially as the condensed water molecules can give rise to capillary forces [101]. Ronkainen *et al.* [108] evaluated the tribological performance of different DLC films in water-lubricated conditions. Their results showed that the amorphous hydrogenated carbon films could not survive in the water-lubricated conditions, and was worn through during the test, while the hydrogen-free DLC films fabricated by vacuum arc discharge exhibited the best wear resistance. However, the wear resistance of hydrogenated DLC films can be improved by doping with Si, W and Cr or by interlayers [108].
C Surface roughness: Surface roughness of the DLC films and its underlying substrates has a decisive influence on the wear of the counterface, especially in the case of a soft material such as ultra high molecular weight polyenthylene (UHMWPE). It was reported that even single scratches in the film, which may be undetectable by an average surface roughness measurement, are capable of increasing the wear rate of UHMWPE by a factor of 30–70 [109]. The effect of the substrate surface roughness on the wear behavior of DLC films was investigated on a ball-on-disk wear rig in dry air by Jiang *et al.* [110]. The wear rate of the films increased significantly with the increase in the substrate surface roughness, while the frictional behavior was not apparently affected. Roughness of 0.93 μm was found to be the critical substrate surface roughness, above which the dominant wear mechanism changed from adhesion to chip/flask formation and fragmentation [110].
D Film thickness: Thick films are preferred for protecting metal from corrosion and wear. However, the compressive stress limits the maximum thickness of the adhesive films and may cause delamination during wearing. Therefore, various methods are used to improve the adhesion strength and reduce the compressive stress. Firstly, cleaning the surface of the substrates with Ar ion bombardment before film deposition is good for the availability of high interfacial adhesion strength. Secondly, forming a mixed interface between film and substrate in the first stage of deposition can also increase the adhesion strength. Thirdly, doping with metal or non-metal elements to reduce the internal stress of the film is also an effective way to obtain high adhesion strength. It was reported that Si doping could improve adhesion strength and reduce internal stress [111], thus increasing the thermal stability of the film as well as the insensitivity of the coefficient of friction to the humidity [112]. Doping metals such as Ta, W, Ti, Nb and Zr in the hydrogenated DLC film also decreased internal stress and lower the dependence of the friction coefficient of the film on humidity [113]. Fourthly, a multilayer approach using alternate soft layer is another effective way to reduce compressive stress in DLC film. Film fabricated by this method showed good friction and wear performance [114]. Finally, diamond-like nanocomposite (DLN) film. DLN film is a new class of materials with reduced compressive stress and increased adhesion strength. This kind of film is composed of two interpenetrating amorphous random network, one is a DLC (α-C:H) network and the other is a glass-like α-Si:O network [113]. Its advantages also include higher temperature stability and a low coefficient of friction.

Although many good results have been obtained on the wear performance of DLC films, some contradictory results are also reported by researchers. In the review of [115], Roy *et al.* gave two examples. Firstly, clinical tests of DLC-coated vascular stents revealed that the DLC film did not provide significant improvements in restenosis rate over uncoated stents. Secondly, ten-year follow-up of DLC-coated artificial hip joints showed that failure rate of the DLC-coated Ti-6Al-4V femoral head was much higher than alumina femoral head. These controversies should be further discussed and more *In vitro* and *In vitro* studies should be done before clinically used on biomedical devices.

## 3. Biocompatibility and Bioactivity

Biomedical implant materials are expected to be biocompatible, bioactive, non-toxic and should not cause any inflammatory or allergic reaction. Biocompatibility was defined as the “acceptance of an artificial implant by the surrounding tissues and by the body as a whole” [116]. For some specialized biomaterials, biocompatibility also includes adequate mechanical properties, appropriate optical properties and suitable density [116].

According to the European Society for Biomaterials consensus conference of 1987, a bioactive material is “one which has been designed to induce specific biological activity”. Upon implantation in human body, bioactive materials are capable of inducing the formation of bony tissue around the implant material and strongly integrating with the implant surface, which is called osseointergration. For bone-bonding materials, bioactive materials are those can induce bone-like HA formation both *in vitro* and *in vivo* [117,118]. Since this review is dealing with the biomedical coatings for hard-tissue application, the term of bioactivity present in the review represents their bone formation ability.

Both biocompatibility and bioactivity of a biomaterial are strongly dependent on its surface properties because cascades of biological reactions occur firstly and directly on its surface as soon as it is fixed into a body [119]. Thereafter, surface properties of an implant, such as surface topography, surface chemical and physical properties as well as surface roughness, will influence the performance of the implant. Recent progresses on coatings for improving the biocompatibility and bioactivity of metallic implants are reviewed below.

Calcium phosphates are the most important inorganic constituent of biological hard tissues. Comprehensive overviews of the basic science and significance of calcium phosphate as biomaterials were given by Paital [120], Dorozhkin [121] and Bohner [122]. Table 3 derived from [121] lists properties of the biologically relevant calcium orthophosphates. So far, only two compounds (*i.e.*, hydroxyapatite (Ca_10_(PO)_6_(OH)_2_) and tricalcium phosphate (α or β-Ca_3_(PO_4_)_2_)) have been extensively tested both *In vitro* and *in vivo* [123]. HA is biocompatible and bioactive in the human body due to its similarity to the mineral component of natural bone. It can adhere directly to osseous, soft, and muscular tissue without an intermediate layer of modified tissue. Various surface modification methods have been applied to fabricate Ca-P coatings, including dip and immersion coating [124], electrophoretic deposition [125], hot isostatic pressing [126], laser deposition [127], thermal spraying (including plasma spraying [128], high-velocity oxy-fuel combustion spraying [129], solution deposition [130], biomimetic coating [131,132] and sol-gel coating [133,134]. Detailed descriptions and comparisons of these methods can be found in the review articles [123,135]. Table 4 lists some characteristics of those often-used surface techniques. Amongst these surface techniques, plasma spraying is currently the most favorable method commercially available for coating biomedical implant devices with HA. Plasma sprayed HA coatings and those influencing factors in their biocompatibility and bioactivity are discussed as follows.

### 3.1. Hydroxyapatite (HA) Coatings

**Table 3:** Properties of the biologically relevant calcium orthophosphate. (Reproduced with permission from Prof. Epple, M. [121])

Ca/P ratio	Compound	Formula	Solubility at 25^o^C, –log(K_sp_)	Solubility at 37 ^o^C, –log(K_sp_)	pH stability range in aqueous solution at 25 ^o^C
0.5	monocalcium phosphate monohydrate (MCPM)	Ca(H_2_PO_4_)_2_·H_2_O	1.14	no data	0.0–2.0
0.5	monocalcium phosphate anhydrate	Ca(H_2_PO_4_)_2_	1.14	no data	[d]
1.0	diacalcium phosphate dehydrate (DCPD, “brushite”)	CaHPO_4_·2H_2_O	6.59	6.63	2.0–6.0
1.0	diacalcium phosphate anhydrate (DCPA, “monetite”)	CaHPO_4_	6.90	7.02	[d]
1.33	octacalcium phosphate (OCP)	Ca_8_(HPO_4_)_2_(PO_4_)_4_·5H_2_O	96.6	95.9	5.5–7.0
1.5	α-tricalcium phosphate (α-TCP)	α-Ca_3_(PO4)_2_	25.5	25.5	[b]
1.5	β-tricalcium phosphate (β -TCP)	β-Ca_3_(PO_4_)_2_	28.9	29.5	[b]
1.2-2.2	amorphous calcium phosphate (ACP)	Ca_x_(PO_4_)_y_·nH_2_O	[c]	[c]	[c]
1.5–1.67	Calcium-deficient hydroxyapatite (CDHA)	Ca_10-x_(HPO_4_)_x_(PO_4_)_6-x_(OH)_2-x_ (0 < x < 1)	≈ 85.1	≈ 85.1	≈ 6.5–9.5
1.67	hydroxyapatite	Ca_10_(PO_4_)_6_(OH)_2_	116.8	117.2	9.5–12
2.0	tetracalcium phosphate (TTCP)	Ca_4_(PO_4_)_2_O	38–44	37–42	[b]

The solubility is given as the logarithm of the ion product of the given formulae (excluding hydrate water) with concentrations in mol/L. [b] these compounds cannot be precipitated from aqueous solution). [c] cannot be measured precisely. However, the following values were reported: 25.7 ± 0.1 (pH 7.40), 29.9 ± 0.1 (pH 6.00), 32.7 ± 0.1 (pH 5.28). [d] Stable at temperatures above 100 °C. [e] Always metastable. The composition of a precipitate depends on the solution pH values and composition [121].

Plasma sprayed HA coating was first used for improving the fixation between bone and implants in 1980s [136] and the clinical trials of HA coatings first used in the femoral stem was by Furlong *et al.* in 1985 and was reported in 1991 [137]. Since then, HA coatings have been extensively studied and their applications have extended to coat acetabular components, knee prosthesis, pin/screw components and dental implants [128]. The quality of HA coatings is strongly depend on their fabrication methods and can be characterized by the following specifications: crystallinity, thickness, phase composition, surface roughness, microstructure and porosity, among which microstructure, crystallinity, surface roughness and phase composition are of great importance to their biocompatibility and bioactivity.

**Table 4:** Summary of surface techniques for depositing Ca & P coatings on metal implants and their characteristics.

Methods	Characteristics
Dip and immersion coating	High temperature for post-sintering HA layer can degrade the strength of the metal and impair the interfacial adhesion and cause the decomposition of HA
Electrophoresis deposition	Low bond strength and non-uniform thickness of the coating
Hot isostatic pressing	Difficult to seal borders on implants with complex shapes, high temperature during the process may denature HA
Solution deposition	A low temperature deposition method resulting in a pure, highly crystalline, firmly adherent coating
Sputtering deposition	A line-of-sight technique with low deposition rate and high cost, but the coatings are dense and with uniform thickness on flat substrates
Thermal spraying	A line-of-sight technique with high deposition rates and low cost; high temperature may cause decomposition of HA; high cooling rate may result in the formation of nanostructure, coatings usually have micro-rough surface
Sol-gel	Not a line-of-sight technique suitable for coating substrates with complex shapes; processing temperature is low; raw materials are expansive and sometimes including organic toxic solvent.
Biomimetic coating	Low processing temperature technique capable of coating complex-shaped substrates; time-consuming
Laser deposition	Be capable to restore complex stoichiometries and to produce crystalline and highly adherent coatings, but process temperature may cause the oxidation of metal or alloy substrates.

A Crytallinility: Crystallinity of plasma sprayed HA coating varies from 50% to 90%. Currently, there is no agreement on what the optimum crystallinity should be. However, it is generally agreed that HA coatings with low crystallinity have higher tendency to dissolve in the body fluid thus giving rise to a faster bone growth rate compared to those with high crystallinity. However, high dissolution rate of the HA coating may lead to mechanical degradation, deterioration of the interfacial adhesion, which would finally lead to the loss of the fixation and delamination of the coating. In addition, the debris from the coating may cause undesired inflammatory reaction, thereby compromising the fixation of the implant to bone [3].
B Phase composition: High temperature process of plasma spraying usually causes the decomposition of certain amount of HA phase into an amorphous and tricalcium phosphate (α and/or β-TCP), tetracalcium phosphate (Ca_4_P_2_O_9_; *i.e.*, TTCP) and calcium oxide (CaO). The dissolution rates of these decomposition products are much higher than that of HA, and are in the order of TTCP >> α-TCP > β-TCP >> HA [128]. The fast dissolution of these Ca & P compounds can easily produce supersaturated environment for precipitation of apatite on the coating surface, leading to an enhanced bone growth. It should be stressed that calcium oxide is not biocompatible and should be avoided although it has a high dissolution rate [128]. The side effect of the decomposition of HA is that the fast dissolution of the newly formed Ca & P compound may cause the undesirable fast degradation of the coating. Therefore, both the cystallinity and phase composition should be well designed or controlled for the biomedical use of HA coatings.
C Microstructure and porosity: Microstructure and porosity of the HA coatings, depending on the process parameters, particle size and size distribution of the feedstock powders, can control the specific surface area of the coating thus influencing the physiochemical interactions at the implant-host interface [138].
D Surface roughness: Surface roughness of the HA coating has a significant effect both on the initial mechanical stability of fixation and on osteo-integration. Plasma sprayed HA coating has a roughness of several micrometers, which is strongly influenced by the spray parameters [138], such as spray distance, spraying current, plasma forming gases, and powders conditions. Evidence suggests that rougher surface exhibits a greater mechanical fixation with the nature bone as they are more capable to enhance the adhesion of osteoblast cells and their subsequent proliferation and differentiation [139,140]. The proposed mechanism was that rough surface could induce the release of growth factors and cytokines in the adhering osteoblasts [141]. Additionally, rough surface also favors the precipitation of apatite. Firstly, high roughness allows a large contact area between the coating surface and the body fluid, resulting in an increased Ca & P release. Secondly, rough surface provides more nucleation sides with lower interface energy for bone-like apatite to anchor [142].

A major concern of the plasma sprayed HA coating is its low bonding strength to metal or alloy substrates due to the mismatch of thermal expansion coefficients between HA (13.3 × 10^-6^ K^-1^) and substrates (Titanium: 8.4-8.8 × 10^-6^ K^-1^; Co-Cr alloy: 16 × 10^-6^ K^-1^). Interlayer like Ti can somewhat reduce the mismatch of thermal expansion coefficients between HA and substrates thus increasing their bonding strength [143]. In addition, HA coatings are generally brittle with low fracture toughness. Evidence showed that HA composites with TiO_2_ [144,145], Al_2_O_3_ [146,147] and ZrO_2_ [148,149] can overcome this mechanical shortcoming to a certain extent. Recently, it was reported that the introduction of nano carbon tubes could improve the mechanical properties of HA coating [150,151,152,153] without undermining its biocompatibility [152].

Besides plasma spraying, some thin film techniques have also been used to deposit calcium phosphate films. Pulsed laser deposition (PLD) is recently extended to produce calcium phosphate coatings on biomedical substrates. PLD has some advantages over other physical surface modification methods, such as the stoichiometry of the target can be retained in the deposition films; multilayered film can be deposited with a laser beam by simply changing target materials using a rotational multi-target holder; lower temperature is needed to deposit the films; the crystallinity can be well controlled [153].

A typical PLD process is as follows: a pulsed laser beam is focused onto the surface of the rotating target situated inside a vacuum chamber; with the laser radiation interaction, the target material is dissociated and ablated out; the ablated species are deposited onto the substrates surfaces [153,154]. Compared to plasma spraying technique, PLD is able to deposit films with similar chemical composition to the target material due to the flash vaporization resulted from the rapidly transferred energy from the laser beam, which makes PLD a promising technique for coating HA onto metal substrates.

Just like plasma sprayed HA coatings, the bioactivity and biocompatibility of HA films deposited by PLD, are strongly dependant on the crystallinity and chemical composition of the coated material. Gas environment and substrate temperature are the two main influencing factors on the crystallinity and on the Ca/P ratio of HA films deposited by PLD techniques. It was reported that if the temperature of the substrate is below 400 °C, amorphous HA films will be produced [155]. Increasing substrate temperatures is beneficial for producing HA coating with a high crystallinity. However, high temperatures may result in the formation of non-stoichiometries HA films [156,157], which was ascribed to the re-evaporation of phosphorus during the deposition process [158]. Gaseous environment also plays an important role on the formation of crystallized HA films. In an inert gaseous environment, amorphous HA films were produced at substrate temperatures between 400 °C and 600 °C. With the presence of water vapor in the gaseous environment, crystalline HA films were formed [154]. However, the pressure of the water vapor should be accurately controlled. Fernández-Pradas *et al.* [159] found that the water vapor pressure should be near 0.5 mbar in order to get a highly crystalline HA films for both 193 nm and 248 nm excimer laser wavelengths. The water vapor pressure also has great influence on the Ca/P ratio of the HA films. With KrF excimer wavelength and laser energy density of 3.53 J cm^-2^, TTCP (Ca/P = 2) was found in the HA films if the water vapor pressure was below 50 Pa, while α-TCP was formed when the pressure was above 50 Pa. Laser energy density can affect the crystallinity of the HA films [160,161]. It was reported that HA films fabricated at a laser energy density of 3 J cm^-2^ exhibited 98% crystallinity after annealed at 400 °C, while those at a laser energy density of 9 J cm^-2^ possessed only 87% crystallinity after subjected to the same annealing process. Besides gaseous environment, substrate temperature, laser energy density and water vapour pressure, the quality of the PLD HA films is also influenced by laser wavelengths [162] and pulse repetition rate [163,164].

The main drawback of HA films fabricated by PLD is the splashing or the particulates deposition on the film which may roughen the film surface. Studies showed that particulate deposition can be reduced or avoided by using short wavelength laser [165], or a mechanical particle filter which can remove slow-moving particulate [154], or by using a sintered HA target with a high density and a defect-free smooth surface [154].

**Figure 2:**
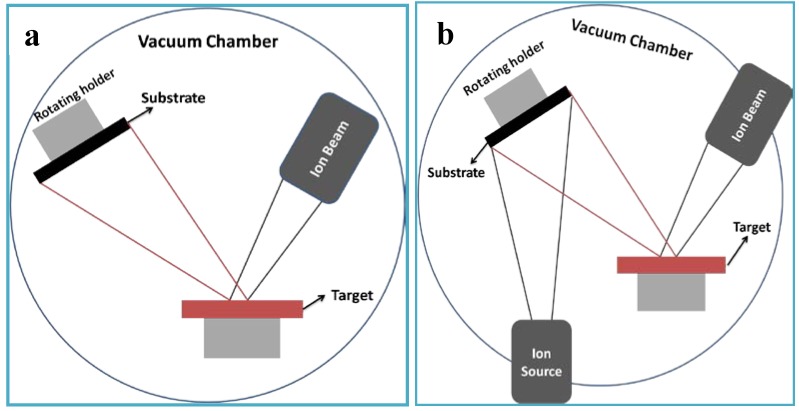
Schematic maps of typical IBSD and IBAD process (a) IBSD. (b) IBAD.

Ion beam techniques, such as ion beam sputtering deposition (IBSD) [166,167,168,169], ion beam assisted deposition (IBAD) (Figure 2) [168,170,171] have also been widely used to deposit calcium phosphate thin films on the metallic substrates. These techniques can produce a thin, homogeneous calcium phosphate with high adhesive strength. In IBSD process (Figure 2a), metal substrates are first fixed on a rotating stage located in the vacuum chamber. After a minimum base pressure is obtained, high purity argon is backfilled the chamber to a pressure with which the deposition is accomplished. Then, substrates precleaned by Ar-sputtering are coated with ions sputtered from the targets. Ong *et al.* [172] deposited thin amorphous HA films on titanium substrates with HA-fluorapatite sintered target. The bonding strength of the as-sputtered films is 38.0 ± 8.2 MPa.

Figure 2b shows the typical process of IBAD. The differences between the IBSD and IBAD are that the latter is combined with ion beam bombardment. The most important advantage of IBAD over IBSD is that a higher adhesive strength can be obtained because an atomic intermixed interface is formed by the ion bombardment [168,170,173]. Hamdi *et al.* [170] prepared HA films by using ion beam deposition assisted by an Ar ion beam with preheated CaO and P_2_O_5_ powder as Ca and P precursors. It was proved that the Ca/P ratio of the films strongly depended on the ion beam current density, indicating that the chemical composition is controllable by changing the processing parameters. Cui *et al.* [168] compared the adhesive strength of the HA films deposited by IBSD and IBAD techniques. The target they used was composed of 70% HA and 30% tricalcium phosphate (Ca_3_(PO_4_)_2_, TCP). The bombardment energy of Ar^+^ beam used to produce an atomic intermixed interface between film and substrate was 30keV, while that for assisting coating growth and reinforcing coating compactness was 200eV. The adhesive strength of IBAD coating was nearly twice that of IBSD coating. Using energetic Ca^2+^ ion beam, HA films with high adhesive strength can also be obtained [172,174,175,176]. Besides high adhesive strength, low substrate temperature, high reproducibility, and controllability over microstructure and chemical composition also make IBAD attractive for coating metallic substrates with HA [170].

The main drawback of the HA films fabricated by ion beam techniques is their amorphous phase composition which leads to high dissolution rate in biological fluids [167,177]. To increase the crystallinity of HA films, heat treatments at 500 °C–600 °C are often utilized [166,172]. However, heat-treatment has some negative effects on the adhesive strength of HA to metallic substrates. Even so, the adhesive strength of HA films deposited by ion beam technique is higher than those fabricated by using plasma spraying [176].

Magnetron sputtering deposition is anther effective way to produce HA films on biomedical implants. The process is similar to that of ion beam sputtering deposition. Briefly, when powder is supplied to a magnetron, a negative voltage is applied to the target. With this negative voltage, positive ions can be attracted to the target surface, leading to the collision with the target surface at the atomic level. Then, atoms sputtered from the target conform to the substrate surface and form a film. Distinctly, a magnetron field is used in the magnetron sputtering process in order to confine the secondary electron close to the target, thus increasing the collision rate of sputtering ions with the atoms of the targets [178,179]. To enhance the adhesive strength of magnetron-sputtered HA films with metallic substrates, composite HA coating with Ti [180] and functionally graded HA/Ti films [178] have been fabricated. In addition, using TiN interlayers between the HA films and substrates can also increase the adhesive strength [181]. For improving the cytocompatibility of the HA films, bioactive elements like Si are introduced to HA films with magnetron co-sputtering deposition technique [182]. Other thin film techniques, such as hot isostatic pressing [126,183], biomimetic methods [131,132,184,185], and sol-gel method [133,134,186,187] can also be utilized to deposit HA films on metallic substrates.

### 3.2. Bioactive Glass and Glass-ceramics Coatings

Besides those mentioned above, another important branch in bioactive coating family is CaO-SiO_2_ based materials, namely bioglass, bioceramic, and glass-ceramic. Since Hench discovered bioglass in 1969 [188], these CaO-SiO_2_ based materials have been extensively studied. Their excellent bioactivity and well-documented biocompatibility make them ideal for biomedical applications, particularly in orthopaedic and dental implants.

Bioactive glasses and glass-ceramics can indeed elicit complex, multi-stage interactions with living body fluids and living tissues, whereby the surface of the component undergoes chemical and structural alterations which subsequently favour the growth of bone tissues [117]. The glassy network of these materials can be partially dissolved by body fluids, releasing Ca^2+^ and P^5+^ ions and forming large amounts of bioactive Si-OH groups. Si-OH groups on the coating surface are beneficial for the nucleation and growth of apatite in the body fluids which is supersaturated with respect to HA [189], thus leading to the formation of a surface layer with a chemical and structural affinity to bone tissues. Si ions released from bioglass can stimulate intracellular reactions and further assist the bone tissue in bonding to the surface of bioglass [190,191,192].

Surface techniques for producing bioglass coatings include plasma spraying [193], high-velocity suspension flame spraying [190], sol-gel [192,194], enameling technique [195], electrophoretic deposition [196], ion beam sputtering [197], laser cladding [198], and pulsed laser deposition [199]. Plasma spraying and sol-gel coating techniques are used more frequently. Interfacial bonding is a main concern involved in the plasma sprayed bioglass coating due to the mismatch of thermal expansion coefficients between the glass and the underlying metallic substrates. Compositing with HA [200], and properly adjusting SiO_2_ amount [201] can improve the bonding strength of bioactive glass to the metallic implants. Sol-gel is an alternative method for coating bioglass on metallic implants by which bioglass coating is fabricated at much lower temperatures than those traditional methods require. Sol-gel derived bioglass has a number of advantages over melt-derived glass, including purity, homogeneity, higher rate of hydroxyl-carbonate apatite (HCA) layer formation and resultantly rapid bone fixation [192]. Additionally, the bioactive range in the CaO-SiO_2_-P_2_O_5_ is larger for sol-gel materials than that for the corresponding glass obtained by melting [202].

**Figure 3:**
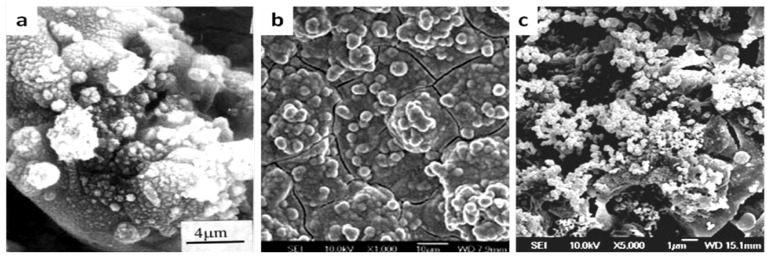
Comparison of bone-like apatite formation on plasma sprayed CaO-SiO_2_ based bioceramic coatings after immersion in SBF solution: (a) CaSiO_3_ coating immersed in SBF solution for 1 day. (b). Ca_2_SiO_4_ coating immersed in SBF solution for 1 day. (c) CaMgSi_2_O_6_ coating immersed in SBF solution for 5 days. (Figure 3a and b are reprinted with the permission from Liu, X.Y. [204,205]; Figure 3c is reprinted with the permission from Xue, W.C. [207])

Recently, CaO-SiO_2_ based bioceramic coatings, such as wollastonite (CaSiO_3_), dicalcium silicate (Ca_2_SiO_4_) and diposide (CaMgSi_2_O_6_), have been widely studied by Liu and Xue *et al.* [203]. Figure 3 displays the surface morphologies of plasma sprayed CaO-SiO_2_ based bioceramic coatings after immersion in SBF solution. Bone-like HA is formed on the surface of plasma sprayed CaSiO_3_ coatings after immersion in SBF solution for 1 day (Figure 3a) [204,205]. For plasma sprayed Ca_2_SiO_4_ coating, some apatite particles were observed on the coating surface only after 1 hour and a dense apatite layer was formed after 1 day (Figure 3b), indicating the superior bioactivity of plasma sprayed Ca_2_SiO_4_ coatings [206]. For plasma sprayed CaMgSi_2_O_6_ coating, 5 days are needed to induce the formation of apatite [207,208], as shown in Figure 3c. The main mechanisms for the bioactivity of the plasma sprayed CaO-SiO_2_ based ceramic coatings can be depicted as follows [209]: Ca ions are firstly dissolved from coatings leading to increased ion activity product of the apatite in the surrounding body fluid. As a result, a Si-rich layer with a large amount of negatively charged Si-OH groups is formed, which is favorable for apatite nucleation and growth. The dissolution rates of these three coatings are in the following ascending order: Ca_2_SiO_4_ > CaSiO_3_ > CaMgSi_2_O_6_. However, the high dissolution rates of these coatings, especially for Ca_2_SiO_4_ and CaSiO_3_, are harmful to their long-term and mechanical stability. However, compositing CaSiO_3_ and Ca_2_SiO_4_ coatings with certain amount of TiO_2_ and ZrO_2_ can improve their mechanical properties without impairing their bioactivity [210,211]. *In vitro* cell experiments showed that osteoblast cells can adhere, proliferate and grow well on these coatings indicating that plasma sprayed CaO-SiO_2_ based ceramic coatings are cytocompatible [206,207,208]. Moreover, Sun *et al.* [212] found that the dissolution products from plasma sprayed Ca_2_SiO_4_ coatings could enhance the expression of osteoblast-related genes and promote differentiation of MG63 cells at the initial period in agreement with the cell responses to bioactive glass, as mentioned above. Additionally, incorporation of biologically relevant trace elements such as zinc [213], strontium [214] and zirconia [215] can enhance the bioactivity and cytocompatibility of Ca-Si based biomaterials [213,214,215].

### 3.3 Bioactive Oxide Coatings

**Figure 4:**
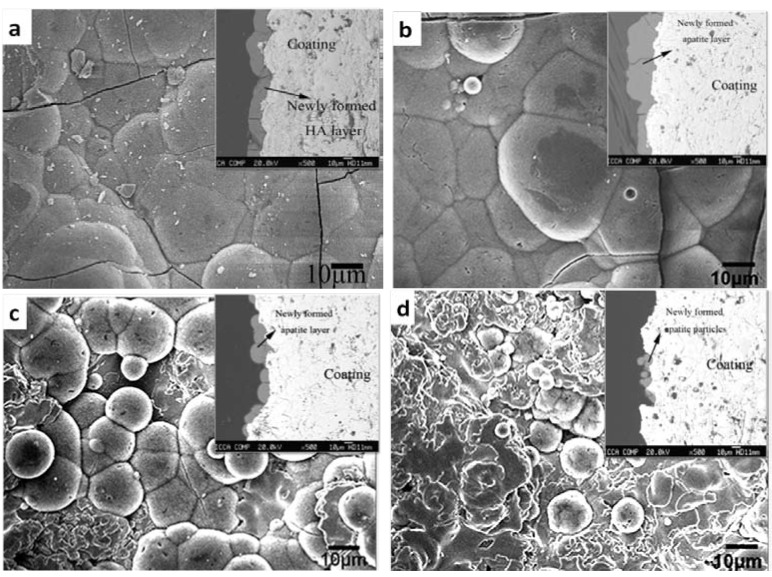
Comparison of bone-like apatite formation on plasma sprayed zirconia coatings after immersed in SBF solution for 28 days. (a) undoped zirconia coating. (b) zirconia coating stabilized 12.8 mol % calcia. (c) zirconia coating stabilized with 16 mol % calcia. (d) zirconia coating stabilized with 30 mol % calcia.

Oxide coatings, such as TiO_2_ [216,217,218], ZrO_2_ [219,220,221] and SiO_2_ [222] also have bioactivity. However, their bioactivity is depending on the coating techniques and the process parameters. A summary of bioactive oxide coatings is given in Table 5. Surface structure (including roughness, nano-sized grains), crystal structure, and surface –OH groups strongly influence the bioactivity of the oxide coatings. –OH groups result in negatively charged surfaces which account for the bioactivity of many sol-gel TiO_2_ and SiO_2_ coatings. However, the function of –OH groups varies according to the crystal structure where the –OH groups are located. For example, it has been proved that the –OH groups on anatase and rutile TiO_2_ is more efficient in inducing apatite precipitation in SBF solution than amorphous TiO_2_ [223]. Additionally, sol-gel alumina coatings do not show bioactivity although they have many –OH groups on their surfaces [222]. It is generally thought that zirconia is a bioinert material because zirconia ceramics scarcely possess the ability to induce bone formation in biological environment. However, recent studies by Wang *et al.* [219,224] and Han *et al.* [225,226,227] demonstrated the *In vitro* bioactivity of the plasma sprayed and micro-arc oxidized zirconia coatings. We previously showed that apatite precipitation on plasma sprayed zirconia coatings was influenced by the amount of dopant (calcia) used to stabilize zirconia. Figure 4 depicts the surface morphologies of the zirconia coating after 28 days of immersion in SBF solution. The undoped zirconia coating exhibited the best bioactivity with a dense, thick and uniform apatite layer formed on its surface, as Figure 4a and its inset show. The bioactivity of zirconia coating stabilized with 12.8 mol % calcia was a little bit weaker, which was reflected in the non-uniform thickness of the apatite layer on its surface, as the inset in Figure 4b shows. As calcia content increased to 16 mol % and 30 mol %, the amount of the newly-formed apatite particles apparently reduced (Figure 4c, d) indicating that their bioactivity becomes worse. These discrepancies in bioactivity were ascribed to their differences in surface micro- and nano-structure and in the phase composition [219,224].

**Table 5:** Summary of bioactive oxide coatings.

Coating	Coating method	Ref.	Post-treatments	Phase	Influencing factors in bioactivity
TiO_2_	Solution precursor plasma spray process	[234]	Chemically treated in 5M NaOH solution at 80 °C	Rutile	Formation of Ti-OH groups
	Sol-gel	[235]	none	Anatase	Surface topography; charge; charge density
		[222]	450 °C, 2 h	Anatase	Abundant Ti-OH groups and negatively charged surfaces
		[223]	Heat-treatment	Anatase	Crystal structure: anatase show more ability to induce apatite formation in SBF than rutile
	Plasma spraying & plasma immersion ion implantation (PIII)	[236]	Hydrogen incorporation by PIII	Rutile (bulk) & anatase (surface)	Combination of nanostructure and hydrogen incorporation can endow the coating with bioactivity
	Cathodic electrolytic deposition	[237]	None	Anatase (subcrystalline)	Crystal structure
			Below 300 °C	Anatase	
			Above 500 °C	Rutile	
	Anodic oxidation	[238]	H_2_SO_4_ and Na_2_SO_4_ solutions	rutile or rutile/anatase	Crystal structure: amorphous titania cannot induce apatite formation in SBF solution
			CH_3_COOH and H_3_PO_4_ solutions	amorphous titania	
ZrO_2_	Plasma spraying	[219,224]	None	Tetragonal (CaO-ZrO_2_)	Nanostructured surface; crystal structure
			None	Monoclinic (undoped ZrO_2_)	
	Cathodic arc deposition	[239]	None	Tetragonal (undoped ZrO_2_)	Nanostructured surface
	Micro-arc oxidation	[225,227]	None	Monoclinic and small amount of tetragonal ZrO_2_	Basic Zr-OH group
			NaOH treatment		
		[226]	Ultraviolet (UV) irradiation	Monoclinic and small amount of tetragonal ZrO_2_	
SiO_2_	Sol-gel	[222]	Heat-treatment at 400 °C for 2 h	amorphous silica	Silanol group (Si-OH)

Ultraviolet irradiation is an effective post-treatment method to enhance the bioactivity of TiO_2_ and ZrO_2_ coatings. Its effects on plasma sprayed nanostructured TiO_2_ coating and micro-arc oxidized ZrO_2_ coatings were proved in two separate studies [226,228]. The enhanced bioactivity is due to the abundant Ti-OH or Zr-OH groups generated by photocatalysis effect of n-type semiconductor TiO_2_ or ZrO_2_ exposed to UV irradiation.

### 3.4. Bioactive Composite Coatings with Polymers

HA and Ca-Si based bioglass or bioceramic coatings possess excellent bioactivity and biocompatibility, however, their shortcomings in mechanical properties including brittleness, poor tensile strength and impact resistance, are limiting their uses in many load-bearing applications. Moreover, their surface characteristics arising from surface chemical composition may be not good enough for inducing selective cell adhesion, spreading, proliferation and differentiation. Inspired from the structure of nature bone, biopolymers such as collagen, gelatin, silk fibroin and poly(lactide-co-glycolide), have been used to composite with bioactive inorganic materials such as HA. The resultant composites are mechanically superior to individual component due to the ductile properties of the biopolymer which increase the fracture toughness of the inorganic component [229,230,231,232]. In addition, the composites have superior biological properties compared to the individual constituents as the composites combine their excellent bioactivity and biocompatibility [229,232].

### 3.5. Biological Molecules Incorporated Bioactive Coatings

Besides the surface modification methods mentioned above, the bioactivity, osteoconductivity or osseointegration can also be conferred on metal implants by incorporation of biological molecules, such as extracellular matrix, adhesion factors, growth factors and differentiation factors. This attempt or idea is based on the knowledge that adsorption of biomolecules onto the implant surface is a key process for cell adhesion and growth on biomaterials and plays a significant role on bone healing [119]. Therefore, immobilization of bioactive growth factors on the surface of the orthopaedic and dental implants are capable of inducing rapids cell functions, including cellular proliferation and differentiation activity, thus accelerating the tissue regeneration. As far as adhesion factors, fibronectin, various laminins and artificial peptides with specific cell signaling sequence like the RGD-sequence are widely investigated. Growth factors and differentiation factors like transforming factor β1 (TGF-β1), insulin-like growth factor (IGF), platelet-derived growth factor (PDGF) and bone morphogenetic proteins (BMPs) are also intensively studied on their osteoinductive functions.

Some concerns regarding the immobilization of bioactive molecules on implants [240]. For example, a misbalance of growth factors has undesirable effects with several adverse side effects; high local doses can be associated with unresolved inflammation. Therefore, it is necessary to appropriately tailor the surface to have a controlled and local release of growth factors. The releasing rate is depending on the surface area of the implants, immobilization methods [241] and the type of carriers used [240].

Generally, the bioactive molecules are immobilized to metal implant surface by adsorption, covalent binding and incorporation in carriers [241]. Table 6 summarizes the different immobilization methods of bioactive molecules. The adsorption can be divided into physisorption and chemisorption, the latter is based on the chemisorptive interaction between the molecules and implant surface. Chemisorption is a useful way to tether/immobilize bioactive molecules to titanium implant surface, the interaction between phosphonate groups and surface oxides are thought to be the main mechanism [242]. Physisorption is based on the electrostatic interactions between the charged surface and the opposite-charged bioactive molecules. The nature of surface charge of an implant can be characterized by the isoelectric point (IEP). If the IEP of a surface is less than the pH value of peri-implant microenvironment, the surface will be negatively charged. In this case, positively charged biomolecules can be adsorbed to this surface. Conversely, the surface will be positively charged and ready for adsorption of negatively charged biomolecules. While the adsorptive method is simple, however, the fixation stability of the biomolecules is not sufficient and their release cannot be controlled [241].

Covalent coupling of bioactive molecules on biomaterial surfaces is an alternative method to adsorption immobilization, which allows a stable fixation of the bioactive molecules. Moreover, the biological activity of the bioactive molecules can be preserved if they are combined with some linker or spacers [243]. For covalent immobilization molecules onto the surface of metal implants, some functional groups should be introduced to their surface prior to covalent immobilization, or by coupling the bioactive molecules with some carriers such as collagen matrices, poly-L-lactide. For immobilization bimolecular on the surface of Ti implants, silanization is often performed before immobilization, to produce a surface with high affinity for some bioactive molecules. Naci *et al.* [244] linked aminoalkylsilane spacer molecules to the surface oxide of Ti implants by heating them in refluxing toluene containing 10% (3-aminopropyl)triethoxysilane, then covalently immobilized alkaline phosphatase or albumin. Results showed that bound proteins were at a biological relevant density and retained their enzymatic activity (alkaline phosphatase) and their antigenicity (albumin) [244]. Tebbe *et al.* [243] applied a similar method to covalently immobilize heparin to Ti substrates and studied the effects of the spacer length on the heparin coupling efficiency and fibrinogen adsorption. They revealed that the long chain of the spacer molecule was beneficial for the covalent attachment of heparin and samples with long chain spacer molecules showed better biocompatibility.

Besides chemical modification, plasma-based surface modification techniques are also effective and economical to create surfaces suitable for covalent immobilization of bioactive molecules. Comprehensive reviews on plasma-based surface modification of biomaterials have been done by other researchers [32,245]. The review written by Siow *et al.* [245] specially focused on plasmas that generate surfaces with chemically reactive groups by which covalent immobilization of bioactive molecules can be realized. For detailed information, the reader needs to refer to these two review papers [32,245].

With the use of plasma techniques, functional groups like carboxyl, hydroxyl, amine and aldehyde can be introduced to the surfaces of biomaterials. Surfaces containing these groups have biocompatibility and well-established chemical reactions for grafting bioactive molecules such as enzymes, antibodies, proteins, and glysosaminoglycans [245]. Usually, these functional groups can be introduced to surfaces of biomaterials by two ways: plasma treatment in proper gases (e.g., O_2_, N_2_, NH_3_ and CF_4_) and plasma polymerization of monomers containing the desired groups. Take amine groups for example, they can be formed on the metal surfaces by both plasma treatment with ammonia [246,247] and plasma polymerization of alkylamine [248,249]. Puleo *et al.* [250] successfully immobilized bioactive bone morphogenetic protein-4 (BMP-4) on titanium alloy using plasma polymerization of allyl amine. After the plasma polymerization, two-step scheme was used to immobilize protein. Briefly, before immobilization of protein, the amino groups were firstly converted to carboxyl groups by immersion aminated samples in 4% succinic anhydride at room temperature overnight, and then samples were treated with a solution of 1-ethyl-3-(3-dimethylaminopropyl)carbodiimide (EDAC) and N-hydroxysuccinimide (NHS) in a 2-(N-morpholino)ethanesulfonic (MES) buffer. Results showed that the two-step carbodiimide immobilization scheme could retain the activity of BMP-4 [250].

For some applications, a sustained release of bioactive molecules over a long time is required. To achieve this goal, researchers are attempting to incorporate the bioactive molecules into organic coatings such as collagen [258], poly(D,L-lactide) (PDLLA) [257], poly(lactide-co-glycolide) (PLGA) [259] and ethylene vinyl acetate (EVAc) [260]. Schmidmaier *et al.* [257] used BMP-2 to modify the Titanium Kirschner wires with Poly (d,l-lactide) as a carrier, aiming at tailoring the implant surface to have a controlled, local release of growth factors. X-rays demonstrated an almost completely consolidated fracture, biomechanical testing showed a significantly higher maximum load and torsional stiffness, and histological and histomorphometric analyses demonstrated progressed remodeling for samples with IGF-1 and TGF-β1, compared to those without IGF-1 and TGF-β1.

In summary, both chemical modification and plasma-based modification can be used to immobilize bioactive molecules onto the surface orthopaedic and dental implant. Although plasma treatment and plasma polymerization are very useful for immobilization of bioactive molecules onto biomaterial surface, the substrates used are mainly composed of polymers. Studies on the metal substrates for orthopaedic application are less comprehensive. This section mainly focuses on the modification of metal or alloys for orthopaedic and dental application, more information on biomolecule-based surface modification can be found in reviews [119,241,261,262].

## 4. Antibacterial Coatings

Bacterial infection at the site of implanted medical devices is a serious ongoing problem in the biomedical filed. It was reported that approximately 11200 (4.3%) of orthopaedic implants are infected among the 2.6 million inserted into human body annually in United States [263]. Bacterial infection not only causes serious pains and sufferings to patients but also increases the medical cost. In serious cases, prosthesis has to be removed and revision surgery is required. However, the success probability of revision surgery is reduced due to the higher rate of infection resulting from a longer operation time, increased scar tissue formation, or unrecognized infection at the initial revision operation [264]. Therefore, it is necessary to develop implants with anti-bacterial properties

**Table 6:** Summary of different methods to immobilize bioactive molecules onto the surface of metallic implants.

Immobilization method		Biological molecule	Substrate and pre-treatment	Results	Ref.
Adsorption		Bone morphogenetic protein-3 (BMP-3)	Corundum-blasted Titanium alloy; Hydroxyapatite coated Titanium alloy; Ti coated Titanium alloy	BMP-3 coated samples showed more ability to induce new bone formation compared to those without BMP-3	[251]
Covalent immobilization	by chemical pretreatment	Synthetic receptor binding motif mimicking BMP-2	3-aminopropyltriethoxysilane (APTES) coated Titanium	enhance the rate of bone healing as compared with untreated Ti surfaces	[252]
		Laminin and human epidermal growth factors (EGF)	Silanized TiO_2_-film Silanisation by reaction of GPTS^1^	Significantly reduce the amount of irreversibly adsorbed salivary proteins	[253]
		Heparin	Silanized and oxidized Titanium Oxidization by H_2_SO_4_/30% H_2_O_2_ or annealing at 750 °C; Silanisation by being boiled in APMS^2^ contained toluene soltution	The remaining activity of heparin is depending on the chain length of spacer	[243]
	by plasma-based modification	Fibronectin	Plasma polymerization of HMDSO^3^ on Titanium	Enhanced adsorption of fibronectin	[254]
		BMP-4	Plasma polymerization of allyl amine on Titanium alloy	Surfaces with BMP-4 are initially able to induce ALP activity in C3H10T1/2 cells, long term effect is depending on the concentration of surface amino group	[250]
Incorporation with carriers		Recombinant human BMP-2 (rhBMP-2)	Turned or surface etched Titanium dental implant Absorbable Collagen sponge (ACS)	rhBMP-2/ACS significantly enhances the effect of guided bore regeneration (GBR)	[255]
		BMP-2; insulin-like growth factor-1 and transforming growth factor-β1	Titanium Kirschner wires incorporated with poly(D,L-lactide) (PDLLA)	Significantly accelerate the fracture healing	[256,257]

GPTS^1^: (3-glycidyloxypropyl)trimethoxysilane; APMS^2^: 3-(Trimethoxysilyl)-propylamine; HMDSO^3^: Hexamethyldisiloxane

Implant-associated infections are the results of bacteria adhesion, attendant colonization and the formation of biofilm. The first and most important step for bacteria to interact with the implant is bacterial adhesion on the implant surfaces which is preceded by the adsorption of a conditioning film of small organic compounds and macromolecules [6]. Therefore, inhibiting bacterial adhesion is a crucial step to preventing the implant-associated infection. Based on this knowledge, material scientists have been attempting to incorporate some antibacterial agents to implant surface aiming at inhibiting bacterial adhesion. Anti-bacterial agents for artificial medical implants can be classified into two categories (*i.e.*, inorganic and organic anti-bacterial agent): Organic anti-bacterial agents include antibiotics, such as vancomycin, tobramycin, cephalothin, gentamicin *etc.* [265], and human and humanised antibodies including IgG, IgA, IgD, IgE and IgM *etc.* [266]; Inorganic antibacterial agents include Ag-related agents, TiO_2_, ZnO and carbon films *etc.*

### 4.1. Antibacterial Coatings with Inorganic Agents

Table 7 is a summary of the inorganic agents which have been used or studied to prevent implant-related infections. The antibacterial properties of silver or silver ions and their non-toxicity to mammalian tissue have been known for a long time. The antibacterial mechanism of Ag-related materials has been widely studied [267,268,269]. According to Holt and Bart, silver ions can disrupt the function of bacterial cell membranes, uncouple the respiratory chain from oxidative phosphorylation [265,266], collapse the proton-motive force across the cytoplasmic membrane [270] and interact with thiol groups of membrane-bound enzymes and protein [271]. Also, Ag ions can displace other metal ions that are essential to cell survival, such as Zn and Ca [265].

Ag ions has antibacterial activity against a broad spectrum of pathogens found at the implant sites, including *P. aeruginosa*, *E. coli*, *S. aureus*, and *S. epidermidis* [265]. In order to give implant antibacterial function, many attempts have been made to incorporate Ag ion or nano-Ag particles to implant surfaces with or without carriers. Various surface modification techniques have been applied to incorporate Ag^+^ ions or Ag nanoparticles into biomedical implant surfaces, including sol-gel [272], ion implantation [273], ion beam assisted deposition [274], plasma electrolytic oxidation [275], solution/evaporation [276] and chemical vapor deposition [277]. Indeed, due to the inherent drawbacks of the metal implants like Ti and Ti alloy, most of the current studies are trying to incorporate Ag or Ag compound into an existing coating or film on the implant surface, so that dual function or multifunction can be achieved. For example, Zhao *et al.* [274] fabricated Ag/TiN multilayer coatings using ion beam deposition method and demonstrated that the coatings possessed antibacterial function depending on the modulation period. The antibacterial activity of these coatings is mainly affected by the release of Ag^+^ ions [274,276,278]. Therefore factors such as the existing form of silver, the property of host materials and even the particle size [279] and shape [280] can influence the antibacterial activity of the Ag-related coatings. Lok *et al.* [279] found that only partially oxidized nano-Ag particles exhibited antibacterial activities and the formation of the Ag^+^ on the surface of the nanoparticles was thought to be carriers of chemisorbed Ag^+^ in quantities that are sufficient to mediate antibacterial activities.

**Table 7:** Summary of inorganic antibacterial agents studied in biomedical coating applications.

Inorganic agents		Coatings	Coating methods	Testing bacteria	Note	Ref.
Ag-related agent		TiN/Ag multilayered films	ion beam assisted deposition	*E. coli*	Antibacterial activity is depending on the modulation period	[284]
		Silver doped perfluoropolyether-urethane coatings	Coating /evapration	*P. aeruginosa* *A. baumannii* *S. epidermidis*	Antibacterial activity is depending on the release of Ag ions	[278]
		TiO_2_-Ag coating	Plasma electrolytic oxidation in Ag nanoparticle- contained electrolyte	*S. aureus*	Possibly, antibacterial activity is due to the close contact of bacteria with Ag particles and the release of Ag ions	[275]
		polyethylene terephthalate implanted with Ag ion	Ion beam implantation	*S. epidermidis*	Ag exists in the form of Ag_2_CO_3_ and Ag_2_O	[281]
		Poly(vinyl alcohol) / AgNO_3_	Solution/evaporation	*E. coli* and *S. aureus*	Ag ions can release from the composite coating	[276]
		Silver doped SiO_2_ film	Sol-gel	*E. coli* and *S. aureus*	Reduction of Ag^+^ ion is affected by the annealing temperature	[272]
Non-Ag agent	F	F^-^-implanted titanium	Ion implantation	*P. gingivalis* and *A. actinomycetemcomitans*	Antibacterial activity was supposed to be caused by the formation of a metal fluoride complex on the surfaces	[282]
	C	Carbon film	Plasma sputtering for H-free film Chemical vapor deposition for α-C:H film	*E. coli*	α-C:H film showed relatively poor antibacterial activity compared with hydrogen-free carbon films	[283]
	TiO_2_	TiO_2_ film	plasma source ion implantation followed by annealing	*A. actinomycetemcomitans* *F. nucleatum*	Antibacterial activity is due to the photocatalytic bactericidal effect	[284]
		TiO_2_ film	A flame-assisted CVD to deposit SiO_2_, and thermal APCVD to deposit TiO_2_	*E. coli*		[277]
		DLC films containing TiO_2_ nanoparticles	plasma-enhanced chemical vapor deposition	*E. coli*	Enhanced antibacterial activity are contributed by the increased hydrophilicity and the decreased interfacial energy of bacteria adhesion	[285]
	ZnO	ZnO coated glass	Ultrasonic irradiation	*E. coli* and *S. aureus*	The antibacterial activity is due to the generation of the reactive-oxygen-species (ROS) products	[286]

Dowling *et al.* [287] proved that the addition of small amount of platinum (Pt) enhanced the antibacterial activity of Ag coating as active Pt enhanced the Ag^+^ formation through galvanic action thus favoring the release of Ag^+^. The effects of particles size, shape as well as particle distribution on the antibacterial nano-Ag particle contained coatings were studied by Lok *et al.* [279] and Pal *et al.* [280]. Usually, the smaller the particle size is, the better antibacterial activity the particles show [279]. However, reducing the particle size in itself is not the ultimate aim as the antibacterial activity of Ag nanoparticles is also shape-dependent, as previeously reported [280]. Aggregation of nano-sized Ag particles can also lead to a reduction in, or a loss of antibacterial activities [279].

The antibacterial properties of TiO_2_-contained materials are based on the photocatalytic bactericidal effect. Under ultraviolet A (UVA) illumination, TiO_2_ can decompose various organic compounds and generate active-oxygen species such as reactive-oxygen-species (ROS) products such as super-oxide (O_2_^−^), hydroxyl radical (•OH) and hydrogen (H_2_O_2_) [288]. These decomposition products can kill bacteria in the way that they destroy the outer membrane of bacterial cells, thus causing the cell death [289,290]. The anatase TiO_2_ film fabricated by plasma source ion implantation (PSII) followed by annealing, exhibited a strong photocatalytic reaction under UHA illumination and showed a good antibacterial activity in that the viability of Actinobacillus actinomycetemcomitans (*A. actinomycetemcomitans*) and Fusobacterium nucleatum (*F. nucleatum*) on the film was inhibited to less than 1% under UVA illumination within 120 min [284]. It is documented that nanosized TiO_2_ particles can improve the antibacterial activity of diamond-like carbon films fabricated from plasma-enhanced chemical vapor deposition (PECVD) [291]. Besides the photocatalytic effect, the increased hydrophilicity and the decreased interfacial energy of bacteria adhesion contributed to the enhanced antibacterial activity of TiO_2_-contained DLC films.

ZnO is another inorganic antibacterial agent that widely used in the dental cements [292,293]. It shows a marked antibacterial activity in neutral region (pH = 7) even without exposure to light [294]. The anti-bacterial activities of ZnO nanosized powders have been extensively studied. It was reported that the antibacterial activities increased with the decrease in the particle size and the increase in the nanoparticle concentrations. Additionally, the antibacterial activities can be affected by lattice constant (*c_0_*) [295] and crystallographic orientation [296]: Yamamoto *et al.* [295] revealed that the antibacterial activity of ZnO increased with the increase in *c_0_* values; Ohira *et al.* [296] demonstrated that antibacterial activity of ZnO powders with crystallographic orientation was weaker than that of powders without orientation at the same powder concentration. The antibacterial mechanism of ZnO is related to the ROS products such as O_2_^−^, •OH and H_2_O_2_ [297,298], which is similar to that of TiO_2_. However, few attempts so far have been made at utilizing the antibacterial properties of ZnO in biomedical implants.

Carbon films process antibacterial activity to a certain extent depending on the chemical composition of the films [283]. It was reported that H-free carbon films showed approximately two times antibacterial activity than α-C:H films. Besides those inorganic antibacterial agents mentioned above, F^-^ ion implantation can also make titanium antibacterial [254].

### 4.2. Antibacterial Coatings with Antibiotics and Antibodies

The current clinical strategy to prevent implant-related infection with antibiotics is to treat the patient with a high concentration of antibiotic and this is the so-called systemic administration strategy. Major problems associated with this strategy include poor distribution of the antibacterial agent at the implant site due to limited blood circulation to the infected skeletal tissue, and inability to directly address the biofilm pathogen scenario [299]. Although sometimes high dosage of antibiotics can penetrate tissue or biofilms formed on the implants, toxic side effects are also likely to happen in this case. Researchers are now endeavoring to substitute conventional systemic therapy with a controlled and local antibacterial release system [265]. According to the review by Wu *et al.* [299], the advantages of controlled release system over the systemic antibiotic therapy can be summed up in the following several points: administration of low dosage when required, greater control over toxicity and bioavailability of dose, less susceptibility to promoting antibiotic resistance, extended duration of release, avoidance of systemic drug exposure and controlled release from the surface of the implant directly to the site.

An ideal antibiotic release system for treating implant-related infection, like all other drug release systems, should provide an appropriate and effective release profile of the antibiotics at the implant site. Namely, a local and controlled release profile should be ideally characterized by a high initial release rate (burst release) followed by a sustainable therapeutic release over a prolonged periods [6,299]. The most common used antibiotics for treating implant-related infection include vancomycin, tobramycin, cefamandol, cephalothin, carbenicillin, amoxicillin and gentamicin [300]. For controlling the release of antibiotics, the antibiotics need to be doped or loaded in a host material which may be polymers (e.g., polyurethane (PU), silicon rubber, poly L-lactic acid (PLA), poly(glycolic acid) (PGA) and polyhydroxyalkanoates (PHA) and polymethylmethacrylate (PMMA)) and inorganic coatings (e.g., hydroxyapatite). Antibacterial coating developed by this approach is referred to as matrix impregnated coatings. The release of the antibiotics is dependent on the loading amount and physiochemical properties of the antibiotics and the properties of the host materials or matrices.

To attain a controlled antibiotic release system with polymer matrices, the polymers are required to have a controllable hydrophilicity which can be realized by changing the ratio of the hydrophilic and the hydrophobic chains [301] and should also have an appropriate balance of biodegradability and biostability [302]. The hydrophilic polymer allows water molecules to diffuse into the matrices, causing the swelling or/and degradation of the polymers, so that antibiotics can be dissolved in water and be eluted out of matrices, while the hydrophobic polymer chains can retain the stability of the matrices in water. In addition, chemical similarity like lipophilicity between the drug and the polymer matrix can lead to homogenous drug distribution within the polymer, which then influences the release profile of the drug [303].

Basak *et al.* [302] studied the release profile of rifampicin from polyurethane coated implant materials. Polyester urethane coatings with different swelling and degradable properties were synthesized by reacting the ester diol (containing active OH groups) with 2,4-toluene diisocyanate (containing active NCO groups) at different ratio. The swelling of the polyester urethane synthesized from NCO and OH at a ratio of 2.5:1 (PEU2.5) was less than polyester urethane at a ratio of 2:1 (PEU2). The tendency of their degradation behaviors was similar to that of swelling. The release profile of rifampicin from the coatings is characterized by a burst release followed by a sustained release of antibacterial agents. The rifampicin release was dominantly affected by swelling and degradation of the PEU matrices. The burst release, which was supposed to result from that rifampicin bound to the matrix surface and the initial high rate of swelling of the matrix, can help to protect against bacterial adhesion during the most critical period following the implantation [6].

An alternative approach of treating implant-related infections to antibiotic therapy is using immunotherapy with antibodies. Antibodies like IgG, IgA, IgD, IgM and IgE have been proven the most useful as immunotherapeutics [6]. Among them, IgG idiotypes are currently the most attractive antibody therapeutic agent due to their many merits as described in the review paper by Grainger [266]. IgG is a predominant host immune component and is composed of millions of opsonizing antibodies specific to microorganism epitopes. Its antimicrobial or antibacterial activity can be elicited by antiviral binding and neutralization against transfection or by either neutralizing (lethal) binding or opsonisation, promoting F_c_ receptor-mediated phagocytosis and clearance [6,266]. Polyurethane (PU) [304] and carboxymethylcellulose (CMC) [305] hydrogen have been chosen as the delivery matrices for human IgG. In both cases, there was initial burst release of IgG and both of them reduced infections. Briefly, for the controlled system of PU coating, it was proved that *E. coli* adhesion to PU coatings loaded with IgG was significantly reduced compared to those coatings without IgG loading [304]; for the controlled system of CMC hydrogel, results of a mouse model showed that 70% of mice provided with IgG-relasing CMC hydrogen inoculated with *P. aeruginosa* survived after 10 days while there was no survival for mice receiving CMC hydrogen without IgG [305].

Besides polymer coating and hydrogen films, inorganic coatings such as biocompatible HA coatings can also be used as the carrier. Stigter *et al.* used a biomimetic co-precipitation approach to incorporate an antibiotic (*i.e.*, tobramycin) into HA coatings aiming at preventing post-surgical infections in orthopaedic or trauma [306]. Briefly, amorphous calcium phosphate coatings were first deposited onto the Ti6Al4V plates by immersion in 5 times concentrated SBF for 24 nm at 37 °C, followed by immersion in a supersaturated calcium phosphate solution containing tobramycin for 48 h at 37 °C. Drug release test showed that the release of the tobramycin was depending on the coating dissolution, loading dose and pH values at which the test was conducted. The authors contributed the co-precipitation of tobramycin to the chemical structure and the isoelectric point of tobramycin. Antibacterial test proved that this coating was effective against the *S. aureus*. In another study [300], antibiotics with carboxylic groups were found to be better incorporated into HA coatings than those without these groups, which further confirmed the great influence of chemical structure of antibiotics on the co-precipitation of the HA and the antibiotics. In order to achieve a long and sustainable release of the antibiotic, some researchers applied a hydrophobic barrier layer (lipid) to retard desorption of antibiotic [307]. However, the lipid layer may reduce the bioactivity of the implant and can cause side effect on the cell adhesion [306]. In a rabbit infection prophylaxis model, gentamicin-incorporated HA coating, fabricated by combination of an electrochemically assisted process and ink jet technology, was proved to have a high potential to contribute to the improvement of infection prophylaxis in cementless total joint arthroplasty [308]. Compared to the biomimetic method, plasma spraying is a high temperature process and it cannot be used to directly load antibiotics in the coatings. However, certain amount of antibiotics like tobramycin can be adsorbed on the plasma sprayed HA coating due to their affinity to hydroxyapatite. Problems with HA coatings presenting antibiotics on their surfaces are that desorption of antibiotics is rapid and the amount of antibiotics adsorbed on the surface is limited [306].

### 4.3. Nitric Oxide-releasing Antibacterial Coatings

Nitric oxide (NO) is an important regulator and mediator of numerous processes in the nervous, immune and cardiovascular system, including smooth muscle relaxation thus resulting in vasodilation of the artery and increasing blood flow, neurotransmission in the nervous system and macrophage mediated cytotoxicity for microbes and tumor cells [309]. NO can be naturally synthesized in the body by different nitric oxide synthase (NOS). For example, NO can be produced by endothelial nitric oxide synthase (eNOS) under the stimulation of platelet derived factors, shear stress, acetylcholine, and cytokines.

NO can also be synthesized by macrophage during the phagocytosis of bacteria. Exposed to immunostimulants, activated macrophage can produce superoxide (O_2_^−^) and NO which can lead to the formation of a much stronger oxidant peroxynitrite (ONOO^−^) [275]. Peroxynitrite could mediate NO-dependent microbial killing [311]. However, there is a time lag between the production of O_2_^−^ and NO synthesis as the former is immediate but the later needs several hours’ initiation [312]. This time lag time may allow bacteria to create biofilms thus protecting them from the impending ONOO^− ^[310,313]. It is proposed that a supplementary source of NO could favor the ONOO^−^ formation at the initial stage of the colonization when bacteria are most susceptible [310]. Recently, NO-releasing coatings have been proven effective at reducing bacterial adhesion *In vitro* [265,314,315,316] and inhibiting implant-related infections *in vivo* [310,317].

**Scheme 1:**
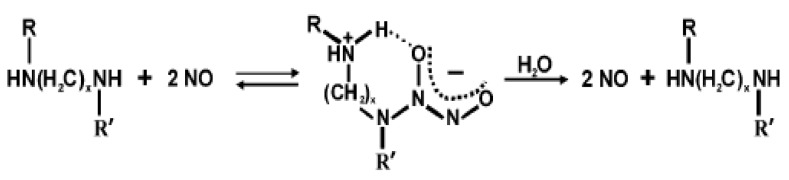
Reaction of NO with amines to produce diazeniumdiolate NO donors [315].

NO donors or functional groups that store and release NO include nitrosamines, organic nitrates, metal-NO complexes, N-diazeniumdiolates, and S-nitrosothiols (RSNOs). Diazeniumdiolate NO donors can be formed by reaction of NO with amines under high NO pressure, as shown in Scheme 1 [315]. When exposed to hydrogen donor, such as water, the diazeniumdiolate decompose to NO and corresponding amine precursors.

Sol-gel chemistry is an easy and effective approach for depositing NO-releasing coatings on biomedical implant surfaces. Briefly, sol is firstly prepared by mixing certain amount of butyltrimethoxysilane (BTMOS), ethanol, water, and hydrochloric acid, followed by adding certain amount of silicon amino-alkoxides (*i.e.*, N-(2-aminoethyl)-3-aminopropylmethyldimethoxysilane (AEAP2), (aminoethylaminomethyl) phenylethylethyltrimethoxysilane (AEMP3), N-(6-aminohexyl)-aminopropyltrimethoxysilane (AHAP3), and (3-trimethoxysilylpropyl)-diethylenetriamine (DET3)) [316]. For depositing coatings on implant surfaces, both drop casting and dip coating methods can be used. Nablo *et al.* deposited NO-releasing coatings on the glass slide and stainless steel substrates using sol-gel methods [310,316,318]. They studied four different amine precursors on their NO releasing behaviors as well as their bacterial activities. A steady rate of NO release was observed for all the sol-gel compositions within 30 min in phosphate buffer solution (PBS), but the level of NO release was dependent on the amount and the type of amine functionality in the films. The release rate increased in the order of DET3 > AHAP3 ≈ AEAP2 > AEMP3. Bacterial adhesion test revealed that these NO releasing coatings could reduce the bacterial (*P. aeruginosa*) adhesion by 30% to 90% depending on the NO flux from the coatings. At a NO flux less than 1 pmol·s^−1^·cm^2−^, these coatings lost their antibacterial activity. To assess their efficacy as antibacterial coatings for orthopedic implant applications, they applied NO-releasing coatings to modify stainless steel substrates [319]. Results showed that sol-gel coatings have a better stability on the stainless steel substrates and significantly decreased the bacterial adhesion. To validate the results of *In vitro* tests, *in vivo* antibacterial activity of NO-releasing xerogel coatings was further evaluated against an aggressive subcutaneous *S. aureus* infection in a subcutaneous animal model (rat) [310]. After 8 days of implantation, the number of infected implants with NO-releasing coatings was reduced by 82%. Histological results showed that capsule formed around the NO-releasing implants had greater vascularity compared to those without NO donor or uncoated control [310]. Besides the advantages mentioned above, Hetrick *et al.* [265] found that NO-releasing coatings may also promote effective device integration into healthy vascularised tissue and diminish foreign body capsule formation.

S-nitrosothiols (RSNO) is another promising NO donor which can be used in implant application. Compared with N-diazeniumdiolates, concerns regarding the formation of potentially toxic by-products are minimal as the RSNO acts as biological transporters of NO in the blood stream [314]. Most of S-nitrosothiols can be formed by the reaction of parent thiols and acidified nitrite (e.g., sodium nitride) [320], which can be called the nitrosation of free thiols. The release of NO from RSNO can be realized by catalytical decomposition by copper (II), copper (I), irradiation with broad-spectrum light， and/or heat *etc.* More information about the biological role of RSNO can be found in [320].

Riccio *et al.* [314] fabricated RSNO-modified xerogel by sol-gel method. The RSNO was introduced by the addition of 3-Mercaptopropyltrimethoxysilane (MPTMS) and methyltrimethoxysilane (MTMOS). In their experiments, MPTMS was hydrolyzed and co-condensed with MTMOS which was a backbone alkylalkoxysilanes. The nitrosation of thiols of MPTMS/MTMOS xerogel was via the reaction with acidified nitrite. Results showed that the coatings were capable of producing NO for up to 2 weeks under physiological conditions. The nitrosated coatings which were capable of releasing NO dramatically reduced both platelet and bacterial adhesion without impairing the fibroblast cell viability, indicating that RSNO-modified coatings have the potential use as biomedical coatings [314]. They also proved that the release of NO from RSNO-modified coatings could be triggered by various ways: thermal RSNO decomposition, catalytical decomposition by copper (II), and irradiation with broad-spectrum light. Among them, thermal RSNO decomposition at 37 °C seems to be the best plausible trigger for coatings used as biomedical implants.

### 4.4. Antimicrobial Peptide Functionalized Films

A more recent utilization of antibacterial agents for biomedical coating application is antimicrobial peptides (AmPs). AmPs generally composed of 15–45 amino acid residues are the effector molecules of innate immunity [321] and are used as a first line of defense against invading pathogens [322]. As AmPs can be produced by insects, fishes, mammalians, and plants, they are often called natural antimicrobial peptide. AmPs have a broad spectrum of antibacterial activity against gram-positive, gram-negative, and multi-drug resistance bacteria [322]. Moreover, they are not likely to cause the bacterial resistance as their antibacterial mechanism is supposed to base on the specific membrane destabilization effect [323,324]. Based on these merits, developing antibacterial coatings with these natural peptides seems very attractive in handling implant-related infections. Table 8 shows some AmPs being used to antibacterially functionalize biomedical films.

**Table 8:** Summary of antimicrobial peptides (AmPs) used to functionalize films and corresponding incorporation methods.

Antimicrobial peptides	Amino acid sequence	Coating methods	Ref.
Defensin	ATCDLASGFGVGSSLCAAHCIARRYRGGYCNSKAVCVCRN	LbL	[326]
Chromofungin	RILSILRHQNLLKELQDLAL	LbL	[327]
Magainin I	GIGLPLHSAGLPGLAPVGGIMLS	SAMs	[328]
Gramicidine A	VGALAVVVWLWLWLW	LbL	[321]
LL-37	LLGDFFRKSKEKIGKEFKRIVQRIKDFLRNLVPRTES	One-pot EISA	[329]
Ponericin G1	GWKDWAKKAGGWLKKKGPGMAKAALKAAMQ	LbL	[322]

**Figure 5:**
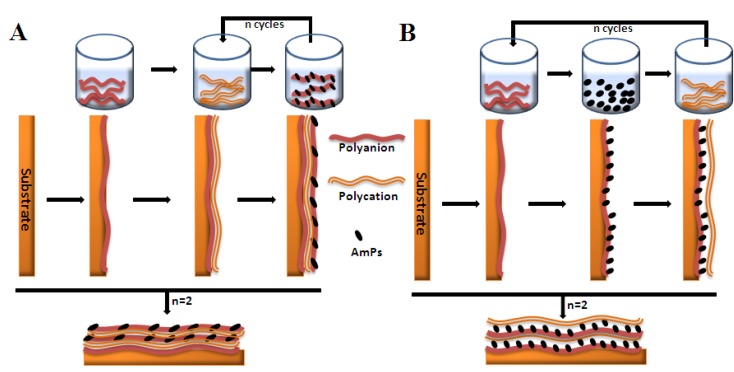
Typical schematic diagram of layer by layer (LbL) methods: (A) The immobilization of peptides is through mixing peptides and the polyanions. (B) The immobilization of peptides is through adsorption onto the pre-existing polyanion layer.

Layer-by-layer (LbL) and self assembly monolayers (SAMs) are two often-used surface methods to embed AmPs into films on solid substrate surfaces. LbL deposition is a simple and powerful means for fabricating multilayered coatings with specified compositions and structures. It is based on alternate deposition of oppositely charged materials on solid surfaces with washing steps in between [325]. The practicability of LbL deposition method on embedding AmPs into films is based on the largely cationic composition of AmPs. In this context, AmPs can be simply incorporated in the polyelctrolyte mutilayers by electrostatic adsorption during film construction or through precomplexation with polyanion used for the LbL assembly [326]. Typical processes of embedding AmPs by LbL are depicted in Figure 5. The immobilization of peptides is either through mixing peptides and the polyanions or by adsorption onto the pre-existing polyanion layers.

Several antimicrobial peptides, such as defensin, chromofungin, gramicidine A, ponericin G1, and LL-37, have been successfully embedded into multilayered polyelectrolyte films by LbL methods, as Table 8 shows. Etienne *et al.* evaluated the antimicrobial and antifungal activity of multilayered films functionalized by defensin [326] and chromofungin [327]. It was proved that both defensin and chromofungin kept their activity after incorporation into polyelectrolyte films. In more detail, the defensin-functionalized films could inhibit the growth of *E. Coli* D22 by 98% [326]; films functionalized with chromofungin was able to inhibit the growth of yeast Candida albicans by 65% and completely inhibit the proliferation of filamentous fungus Neurospora crassa by interacting with the fungal membrane and penetrating into the cells [327]. In the former study, it was observed that the antibacterial activity of the peptides only had effects on those bacteria in close contact with the films for a sufficient time; as a result, the adhesion mechanism was proposed [326]. However, an ideal antibacterial film is required to be not only capable of killing those bacteria attaching on its surface, but also those present around the surrounding tissues or fluids. Therefore, just like other antibacterial coatings, AmPs-functionalized films should also be designed to possess a localized and sustainable release of AmPs to the surrounding meida. Guyomard *et al.* [321] demonstrated that the release of gramicidine A from the amphiphilic polyelectrolyte multilayered films was also active against gram-positive bacteria *E. faecails*. But the release profile was not accurately measured in this work. Shukla *et al.* [322] incorporated ponericin G1 into a hydrolytically degradable polyelectrolyte multilayer film to get a controlled release profile. Three different polyanions (*i.e.*, alginic acid, dextran sulfate, and chondroitin) were used and their effects on film growth, ponericin G1 loading and release were investigated. Dextran sulfate film grew slower than that of alginic acid and chondroiyin due to its much higher molecular weight, as higher molecular species had a slow diffusion process [322]. Accordingly, films with different polyanions possessed different thickness after the same deposition cycles, which directly influenced the ponericin G1 loading amount in the films. Besides film thickness, chemical property is also an influencing factor in ponericin G1 loading and release. The film growth profile and the final thickness of the alginic acid and chondroitin were similar, but their release profile differed from each other as their negative-charged groups interacting with ponericin G1 were different. Antibacterial assay and cell culture test showed that Ponercin G1 could retain its antibacterial activity in all films without compromising their cytocompatibility [322].

Humblot *et al.* [328] immobilized magainins I onto gold surfaces by SAMs. Firstly, the gold-sputtered glass substrates were immersed in a binary mixture of 11-mercaptoundecanoic (MUA) and 6-mercaptohexanol (C6OH). Secondly, the substrates with ad-layers were exposed to a solution of N-hydroxysuccinimide (NHS) and 1-(3-dimethyllaminopropyl)-N’-ethylcarbodiimide hydrochloride (EDC) to convert the carboxylic acid tail groups of MUA into esters function. Finally, magainins were covalently immobilized onto the treated surfaces. The antibacterial activity test showed that more than 50% bacterial cells in contact with the magainins I immobilized surface were killed by modifying the walls of the cell and inducing the formation of pores on outer bacterial membranes. The antibacterial mode was thought to be bacteriostatic [328].

**Figure 6:**
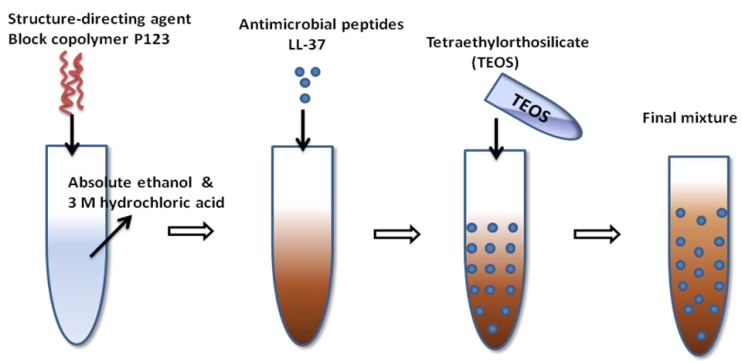
Schematic map of the incorporation of LL-37 into mesoporous silica films by one-pot EISA [329].

Izquierdo-Barba *et al.* [329] utilized a one-pot evaporation-induced self-assembly (EISA) method to incorporate antimicrobial peptide LL-37 into mesoporous silica films and obtained a sustainable release of the LL-37. The incorporation process of LL-37 peptides into the mesoporous silica films is shown in Figure 6. One-pot EISA method is an improved one pot method which runs at a low reaction temperature and suitable for incorporation of highly water soluble proteins and peptides [329]. It was observed that the release of LL-37 from the films was sustainable and the released amount reached the maximum after about 200 h. Moreover, the release rate could be controlled by incorporating SH groups in the pore walls by 3-mercaptopropyltrimethoxysilane (MPTS). Antibacterial activity test showed that LL-37 incorporated film displayed potency against both Gram-positive *S. aureus* and Gram-negative *E. Coli*, indicating its potential use as a biomedical films handling the implant-associated infections.

## 5. Summary

Metallic materials are widely used in biomedical field as orthopaedic and dental implants, as well as cardiovascular devices. However, problems associated with metal implants are limiting their uses and still causing some undesired side effects. Large amount of coatings have been applied to metallic implants with variety of surface modification techniques, however, few studied coatings have multi-functions and few of them have come into clinical use. Therefore, tremendous work should still be done to validate the existing potential coatings and develop new candidates. Multi-functional coatings may be one of the research goals.
